# A newly identified *Leishmania* IF4E-interacting protein, Leish4E-IP2, modulates the activity of cap-binding protein paralogs

**DOI:** 10.1093/nar/gkaa173

**Published:** 2020-03-30

**Authors:** Nitin Tupperwar, Shimi Meleppattu, Rohit Shrivastava, Nofar Baron, Ayelet Gilad, Gerhard Wagner, Mélissa Léger-Abraham, Michal Shapira

**Affiliations:** 1 Department of Life Sciences, Ben-Gurion University of the Negev, Beer Sheva 84105, Israel; 2 Department of Microbiology, Blavatnik Institute, Harvard Medical School, Boston, MA 02115, USA; 3 Department of Biological Chemistry and Molecular Pharmacology, Blavatnik Institute, Harvard Medical School, Boston, MA 02138, USA

## Abstract

Translation of most cellular mRNAs in eukaryotes proceeds through a cap-dependent pathway, whereby the cap-binding complex, eIF4F, anchors the preinitiation complex at the 5′ end of mRNAs and regulates translation initiation. The requirement of *Leishmania* to survive in changing environments can explain why they encode multiple eIF4E (LeishIF4Es) and eIF4G (LeishIF4Gs) paralogs, as each could be assigned a discrete role during their life cycle. Here we show that the expression and activity of different LeishIF4Es change during the growth of cultured promastigotes, urging a search for regulatory proteins. We describe a novel LeishIF4E-interacting protein, Leish4E-IP2, which contains a conserved Y(X)_4_LΦ IF4E-binding-motif. Despite its capacity to bind several LeishIF4Es, Leish4E-IP2 was not detected in m^7^GTP-eluted cap-binding complexes, suggesting that it could inhibit the cap-binding activity of LeishIF4Es. Using a functional assay, we show that a recombinant form of Leish4E-IP2 inhibits the cap-binding activity of LeishIF4E-1 and LeishIF4E-3. Furthermore, we show that transgenic parasites expressing a tagged version of Leish4E-IP2 also display reduced cap-binding activities of tested LeishIF4Es, and decreased global translation. Given its ability to bind more than a single LeishIF4E, we suggest that Leish4E-IP2 could serve as a broad-range repressor of *Leishmania* protein synthesis.

## INTRODUCTION

*Leishmania* parasites cycle between invertebrate vectors and mammalian hosts. In doing so, they differentiate from flagellated promastigotes residing in the intestinal tract of sand-flies, into non-flagellated amastigotes, which are obligatory intracellular forms of the parasites. Amastigotes exist within phagolysosomal vacuoles of macrophages and other cells of the immune system. During their life cycle, a developmental program of gene expression enables the parasites to adapt to different environmental conditions, including temperature, pH and variations in nutrient supplies. Translation regulation plays a key role in driving this program, especially in the absence of conventional transcription activation mechanisms (1–3).

In Opisthokonts, cap-dependent translation initiation is the default pathway for protein synthesis. The translational initiation complex assembles on the 5′ cap (m^7^GTP) of messenger RNAs (mRNAs) through the eukaryotic initiation factor 4F complex (eIF4F). eIF4F comprises the cap-binding protein eIF4E, the DEAD-box RNA helicase eIF4A, and the scaffold protein eIF4G. eIF4G binds eIF3, which recruits the small ribosomal subunit. eIF4G also interacts with eIF4E, through a consensus binding motif, Y(X)_4_LΦ (where X is any amino acid and Φ is a hydrophobic residue). Protein synthesis can be inhibited by the binding of hypo-phosphorylated 4E-BPs to eIF4E. 4E-BP also contains a Y(X)_4_LΦ motif (4) and competes with eIF4G on interacting with eIF4E, thus blocking the formation of the eIF4E/eIF4G complex (5,6). Since the identification of 4E-BP1, many other eIF4E regulatory proteins have been identified in several organisms.

Translation regulation is a central mechanism that drives the developmental program of gene expression in trypanosomatids. This is especially emphasized given their unusual way of generating matured mRNAs (1,7,8). Transcription of primary mRNAs is polycistronic, and there is no evidence for any conventional transcription activation mechanisms of mRNAs. The polycistronic transcripts are further processed to mature monocistronic mRNAs via trans-splicing and polyadenylation (9,10). Since digenetic parasites, such as *Leishmania*, must survive in conditions that affect global translation, cap-dependent and -independent translation mechanisms could be required to generate a stage-specific profile of gene expression. Accordingly, the genomes of *Leishmania* and Trypanosomes encode six paralogs of eIF4E (LeishIF4Es) and at least five paralogs of eIF4G (LeishIF4Gs). These contain a conserved MIF4G domain (11–14) and the consensus Y(X)_4_LΦ element, except LeishIF4G-4 which lacks this motif, despite its strong interaction with LeishIF4E-3 (15). LeishIF4E-1 through −4 were intensively studied both in *Leishmanias* and Trypanosomes (16–21). Two additional orthologs of eIF4E, TbIF4E-5 and TbIF4E-6 were identified in *T. brucei* (22,23), and their orthologs were subsequently found in the *Leishmania* genomes. The high number of eIF4E and eIF4G orthologs in *Leishmanias* and Trypanosomes could coincide with the need of these organisms to survive under extreme conditions at a specific given point during their life cycle. Understanding the roles of these multiple isoforms remains a challenging goal (24).

LeishIF4E-4 is generally accepted to be a canonical translation initiation factor in promastigotes, based on its efficient cap-binding activity and its ability to anchor a functional cap-binding complex, including LeishIF4G-3 and LeishIF4A-1 (25,26). LeishIF4E-4 has a non-conserved N-terminal extension, which contains multiple phosphorylation sites (27). This LeishIF4E-4 N-terminus also binds LeishPABP1, unlike in other eukaryotes, where eIF4G is responsible for this interaction (20,28,29). Exposure of *Leishmania* to mammalian-level temperatures (amastigotes stage) eliminates its cap-binding activity, as well as its binding to LeishIF4G-3. Under these conditions, the isoform LeishIF4E-1 binds efficiently to the cap, suggesting that this protein plays a role in both life stages (20). Unlike these two eIF4E orthologs, LeishIF4E-3 binds inefficiently to the cap-structure, possibly because a Met residue replaces the Trp at position 170 of the protein, which is located within the cap-binding pocket (18). However, the phenotype of a partially silenced LeishIF4E-3 mutant could suggest that it functions in translation (30). The sub-cellular distribution of LeishIF4E-3 is also affected by nutritional stress and was suggested to play a role in storage of inactive mRNAs and ribosomal particles (31). LeishIF4E-3 binds efficiently to LeishIF4G-4, although the latter does not include the conserved Y(X4)LL binding motif (15). LeishIF4E-2 is a polysome-associated eIF4E ortholog that has thus far no identified LeishIF4G binding partner. In *T. brucei*, the LeishIF4E-2 ortholog, TbIF4E-2, associates with a stem-loop-binding protein that binds histone mRNAs (32). Recent data published on two new orthologs of the mammalian eIF4E that were identified in *T. brucei*, TbIF4E-5, and TbIF4E-6, highlighted their eIF4G ortholog binding partners. Silencing of TbIF4E-5 with RNAi suggests that it is involved in cell motility (23).

Given the large number of LeishIF4E paralogs in *Leishmania* parasites and the need to modulate their differential activities, regulatory proteins likely play an important role in the delicate adaptations of the translation machinery during the various stages of the parasite's life cycle. Despite this need, no homolog of 4E-BP or 4E-T could be identified in the genomes of *Leishmania*. Other regulatory proteins have, however, been identified. We recently showed that Leish4E-IP1 is an IF4E-interacting protein that specifically binds the dorsal side of LeishIF4E-1 (20) and that it allosterically destabilizes the binding of LeishIF4E-1 to the 5′ mRNA cap (33). Since the binding of LeishIF4E-IP1 is restricted to LeishIF4E-1, other IF4E-interacting proteins are expected to regulate the assembly of the LeishIF4F complex. Here, we report a newly identified Leish4E-interacting protein, Leish4E-IP2. We found that Leish4E-IP2 contains the consensus IF4E-binding motif Y(X)_4_LΦ, binds several of the LeishIF4E isoforms, and partially affects their cap-binding activities.

## MATERIALS AND METHODS

### Organisms and cell culture

*Leishmania amazonensis* promastigotes were routinely cultured in Medium 199 [M199, (pH 7.4)] supplemented with 10% fetal calf serum (FCS), 5 μg/ml hemin, 0.1 mM adenine, 40 mM HEPES, 4 mM l-glutamine, 100 U/ml penicillin and 100 μg/ml and streptomycin at 25°C. To differentiate promastigote cells into axenic amastigotes, late log phase promastigotes (3.6 × 10^7^ cells/ml) were washed twice with phosphate-buffered saline (PBS) and resuspended in M199, containing 25% FCS, 5 μg/ml hemin, 0.1 mM adenine, 40 mM HEPES, pH 5.5 (adjusted using 0.5 M succinic acid) 4 mM l-glutamine, 100 U/ml penicillin and 100 μg/ml streptomycin. Cells were grown at 33 °C for 4 days.

### Cloning and transfection

We amplified the 1314 bp ORF of Leish4E-IP2 from genomic DNA of *L. amazonensis*, using gene-specific primers (Supplementary Table S1) and cloned it into the BamHI/XbaI sites of the pX-based transfection cassette, pX-H-target ORF-H-SBP (20), where H represents the intergenic region of HSP83 from *Leishmania* and SBP represents a streptavidin-binding peptide affinity tag (34). We transfected *L. amazonensis* cells with the resulting plasmid (40 μg) and selected for resistance with G418 (200 μg/ml). We generated double transgenic cell lines by transfecting constructs allowing the expression of FLAG-Leish4E-IP2 (selected with hygromycin), and of SBP-LeishIF4E-1, LeishIF4E-3 or LeishIF4E-4 (selected with G418).

### Western blot analysis

Wild type *L. amazonensis* promastigotes from different time periods of the growth curve, i.e. early log phase (5 × 10^5^ cells/ml), mid-log-phase (2.6 × 10^7^ cells/ml), late-log phase (3.6 × 10^7^ cells/ml) and stationary phase (4 × 10^7^ cells/ml), as well as axenic amastigotes, were washed twice in PBS (pH 7.4) and once in PRS buffer (35 mM HEPES, pH 7.4, 100 mM KCl, 10 mM MgCl_2_, 1 mM DTT). We resuspended the cells in PRS+ buffer (PRS buffer supplemented with a commercial mix of protease inhibitors (Sigma), 4 mM iodoacetamide, 25 mM sodium fluoride and 55 mM β-glycerophosphate). We lysed the cells by adding Laemmli buffer and boiling the cells at 95°C for 5 min. We resolved equal protein loads by SDS-PAGE followed by western blot analysis with antibodies targeting LeishIF4E-1, LeishIF4E-4, Leish4E-IP1 and Leish4E-IP2. All antibodies were commercially generated against each protein expressed in bacteria. Details of the manufacturers and working dilutions are given in Supplementary Table S2.

### Affinity purification of tagged proteins using streptavidin-Sepharose

We harvested cell extracts (∼10^9^ cells) from cell lines expressing the following SBP-tagged proteins: Leish4E-IP2, LeishIF4E-1, LeishIF4E-3 and LeishIF4E-4. We washed the cells twice with PBS, once with PRS and lysed the cells with 1% Triton X-100 in PRS+, in a total volume of 1.2 ml over a 5 min period, on ice. We centrifuged the extracts at 20 000g for 20 min at 4°C. We incubated the lysates (1.2 ml) with 75 μl streptavidin-Sepharose beads (GE Healthcare) for 2 h and washed three times with PRS+. We eluted the bound proteins with 5 mM biotin in PRS+. Transgenic SBP-tagged Leish4E-IP2, Luciferase and LeishIF4E-1 were purified from cell lysates over streptavidin beads. The eluates that contained their associated complexes were subjected to LC–MS/MS analysis.

### Affinity purification of cap-binding complexes using m^7^GTP-agarose

We harvested wild type (WT) *L. amazonensis* cells and transgenic lines expressing Leish4E-IP2-SBP, washed twice with PBS and once with column buffer (CB) containing 20 mM HEPES, pH 7.4, 2 mM EDTA, 1 mM DTT and 50 mM NaCl. We resuspended the cell pellets in 1.2 ml of CB supplemented with protease and phosphatase inhibitors. We lysed the cells with 1% Triton X-100 in CB with a commercial mix of protease inhibitors (Sigma) and 4 mM iodoacetamide, along with 25 mM sodium fluoride and 55 mM β-glycerophosphate, by incubating on ice for 5 min. We centrifuged the cells at 20 000g for 20 min at 4°C. We incubated the lysate for 2 h with 75 μl m^7^GTP-agarose resin pre-equilibrated with CB. Following binding, we washed the beads with CB containing 100 μM GTP. We eluted the cap-binding complexes with 200 μM m^7^GTP in CB+. We precipitated the proteins with trichloroacetic acid (TCA), at a final concentration of 10%, and resuspended in Laemmli sample buffer. We separated the proteins over 10% SDS-PAGE gels, blotted and probed with specific antibodies. We quantified by densitometry analysis the eluted proteins using MultiGuage 3.0 software. We normalized the quantified proteins in the elution to the total amount of protein initially loaded onto the beads.

### Monitoring the effect of Leish4E-IP2_1-134_ on m^7^GTP cap-binding activity of LeishIF4E paralogs

*Leishmania amazonensis* cells (10^9^) expressing SBP-tagged LeishIF4E-1, LeishIF4E-3 and LeishIF4E-4 were washed twice with PBS and once with CB. Cell pellets were resuspended in 1.2 ml of CB supplemented with protease and phosphatase inhibitors. These included a commercial mix of protease inhibitors (Sigma), 4 mM iodoacetamide, and a mix of phosphatase inhibitors, including 25 mM sodium fluoride and 55 mM β-glycerophosphate. Cells were lysed with 1% Triton X-100 in CB, supplemented with the protease and phosphatase inhibitors, and incubated on ice for 5 min. Cell debris were removed by centrifugation at 20 000g for 20 min at 4°C. The resulting supernatants were split to four aliquots of 300 μl each and incubated with purified recombinant MBP-tagged Leish4E-IP2_1–134_, added in different concentrations (0, 5, 10, 20 μg/300 μl supernatant), for 2 h on a rocking platform. The same protocol was performed for the control experiment, in which the clarified supernatants were incubated with the same concentrations of purified MBP (0, 5, 10, 20 μg/300 μl). The binding mixes were further incubated for two additional hours with 35 μl of m^7^GTP-agarose beads (Jena Biosciences) that were pre-washed with CB. Following this incubation, we washed the beads two times with CB and a final wash was performed with CB containing 100 μM GTP (Sigma). The beads were eluted with Laemmli's SDS buffer and boiling for 5 min. We determined the amount of SBP-LeishIF4Es that were bound to the m^7^GTP agarose beads, in the absence or presence of MBP-Leish4E-IP2_1–134_, by western blot analysis using anti-SBP mouse monoclonal antibodies. The resulting blots were quantified by densitometry analysis using MultiGuage 3.0 software.

### Translation assay

We monitored global translation in wild type cells and in transgenic lines overexpressing a specific target protein using the SUnSET (Surface SEnsing of Translation) assay. This assay is based on the incorporation of puromycin, a tRNA analog, into the A site of translating ribosomes (35). We added 1 μg/ml of Puromycin (Sigma) to cells for 30 min, washed twice with PBS and once with PRS+ buffer. We resuspended the cell pellets in 300 μl of PRS^+^ buffer, denatured in Laemmli sample buffer and boiled for 5 min. We treated the cells with cycloheximide before the addition of puromycin, which served as a negative control. We resolved the samples over 10% SDS-PAGE, followed by western blot analysis using anti-puromycin antibodies.

## RESULTS

### Identification of a potential LeishIF4E interacting protein

We previously identified a *Leishmania* IF4E-interacting protein (Leish4E-IP1) that binds specifically to LeishIF4E-1 (20). We further found that Leish4E-IP1 represses the cap-binding activity of LeishIF4E-1 in promastigotes (36). Despite this progress, it is still unclear how the LeishIF4E-1/Leish4E-IP1 complex relates to the translation process and whether additional proteins are involved in modulating the activity of the different cap-binding proteins in the different *Leishmania* life forms. To address these questions, we sought to identify other LeishIF4E-1 binding partners. We initially analyzed hypothetical proteins that were consistently co-purified with LeishIF4E-1 over affinity columns, for which we used cell lines that expressed LeishIF4E-1 tagged with SBP at its C-terminus (LeishIF4E-1-SBP). We pulled down the transgenic LeishIF4E-1-SBP over a streptavidin-Sepharose resin and submitted the eluted proteins to mass spectrometry analysis for protein identification (Supplementary Table S3). These experiments revealed the presence of a 48 kDa hypothetical protein of unknown function (LmjF33.0380), which contains a typical Y(X)_4_LΦ 4E-binding element between positions 115 and 121 (4). The presence of this conserved motif raised the possibility that LmjF33.0380 could be a direct LeishIF4E-1 interacting protein, it was thus named Leish4E-IP2.

Leish4E-IP2 orthologs were identified in different *Leishmania* species and in trypanosomes. The protein is conserved, but its conservation among *Leishmania* species is higher than among other trypanosomatids, such as *T. brucei* (Supplementary Figure S1), or in the free-living *Bodo saltans*. In the two latter species, the Tyr residue in the 4E-binding motif [Y(X_4_)LL] was exchanged for Phe. Leish4E-IP2 has no orthologs in other eukaryotes and is therefore unique to this group of organisms. Unlike Leish4E-IP1 and other IF4E-binding proteins in Opisthokonts, which are mostly unstructured in their free form, the bioinformatic predictions indicate that Leish4E-IP2 is partially structured (Supplementary Figure S1).

### Leish4E-IP2 associates and co-localizes with different LeishIF4Es

**Figure 1.**
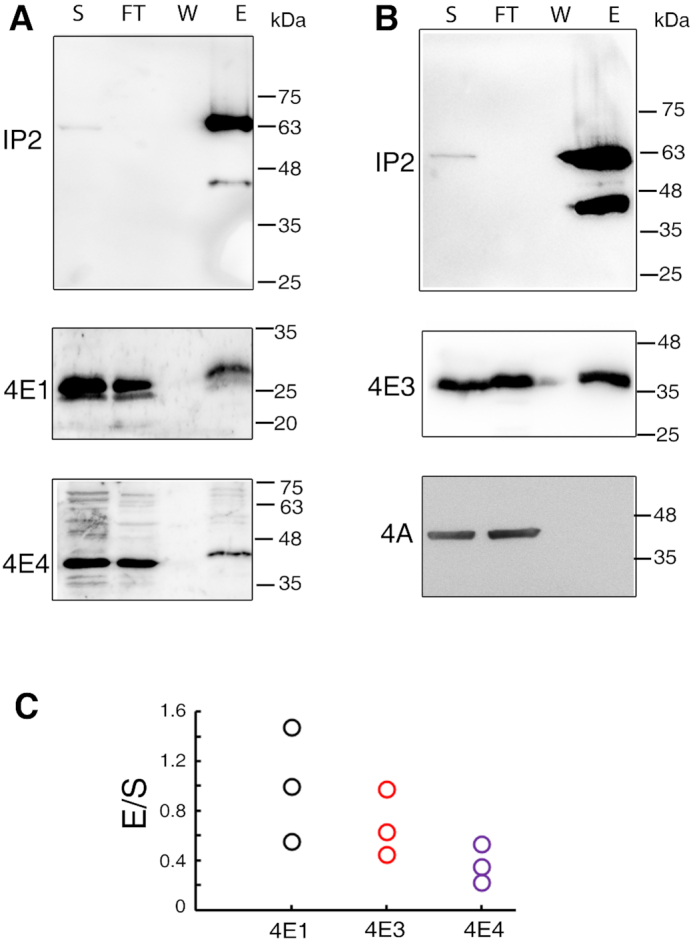
Association of Leish4E-IP2 with different LeishIF4Es. The association between Leish4E-IP2 and LeishIF4E-1 (4E1), LeishIF4E-4 (4E4) (**A**) and LeishIF4E-3 (4E3) (**B**) were monitored by pull-down experiments of mid-log L. amazonensis promastigotes over-expressing SBP-tagged LeishIF4E-IP2. The cells were lysed and affinity-purified over streptavidin-Sepharose beads. The beads were washed and further eluted with biotin. Aliquots from the soluble extract (S, 5%), the flow-through fraction (FT, 5%), the final wash (W, 50%) and eluted proteins (E, 50%) were separated by 10% SDS–PAGE and subjected to western blot analysis using specific antibodies. The antibodies used were raised against LeishIF4E-1, LeishIF4E-4 (A), LeishIF4E-3 and LeishIF4A (as a negative control) (B) along with anti-SBP antibodies that were used to highlight the SBP-tagged Leish4E-IP2. (**C**) Representation of the densitometry analysis of each quantity of LeishIF4E co-eluted by Leish4E-IP2.

We further examined whether Leish4E-IP2 interacts exclusively with the cap-binding protein LeishIF4E-1 or whether it also interacts with the well-studied LeishIF4E-4 paralog. LeishIF4E-1 and LeishIF4E-4 display a different stage-specific profile of cap-binding activities, as LeishIF4E-1 binds the cap both in promastigotes and in axenic amastigotes, whereas LeishIF4E-4 binds the cap-structure mainly in promastigotes (20). We also included in our analysis another paralog, LeishIF4E-3, which is a weak cap-binding protein. Despite this, LeishIF4E-3 is required to maintain translation activity under normal conditions (31) and furthermore, it is involved during nutritional stress. Under such conditions LeishIF4E-3 concentrates in granules that store ribosomal proteins and mRNAs during starvation when translation rates are dramatically reduced (31). We used a DNA construct that allows the expression of Leish4E-IP2 fused to a SBP tag at its C-terminus (Leish4E-IP2-SBP), to transfect and generate a stable *L. amazonensis* cell line. We loaded the cell lysates on a streptavidin-Sepharose resin and eluted the bound proteins using biotin. We analyzed by Western Blots several aliquots taken at the different stages of the purification (Figure 1). Such analysis revealed that Leish4E-IP2-SBP pulled down all the LeishIF4Es that were tested, LeishIF4E-1, LeishIF4E-3 and LeishIF4E-4. LeishIF4A was used as a negative control which was not co-eluted with Leish4E-IP2-SBP. These associations were also confirmed in reciprocal experiments, where LeishIF4E-1-SBP, LeishIF4E-3-SBP and LeishIF4E-4-SBP were able to pull down Leish4E-IP2 (Supplementary Figure S2). The variability of protein bands that interacted with the antibodies raised against Leish4E-IP2 was observed in further experiments along our study, including pull down assays and steady-state level expression. This variability is addressed further on in this manuscript, showing that it represents cleavage products and an unusual migration profile.

To obtain further support for the above-described interactions, co-staining of Leish4E-IP2 with different cap-binding proteins was investigated using confocal microscopy. We used mid-log *L. amazonensis* cells co-expressing a FLAG-tagged version of Leish4E-IP2, and the SBP-tagged versions of the different LeishIF4Es, namely LeishIF4E-1, LeishIF4E-3 and LeishIF4E-4. Co-staining was determined using antibodies directed against the FLAG and the SBP tags, from rabbits and mice, respectively. Secondary antibodies bearing different fluorophores (anti-mouse antibodies labeled with Alexa Fluor 488 and anti-rabbit antibodies labeled with DyLight 550) labeled the different tagged proteins (Supplementary Figure S3). We used the Costes method (37) with BioimageXD software, to quantify the degree of overlap in the co-staining assay, for over 250 cells. Supplementary Figure S3 shows that Leish4E-IP2 co-stained with LeishIF4E-1 (62 ± 18.68%), with LeishIF4E-3 (29.3 ± 15.39%) and mildly also with LeishIF4E-4 (23.0 ± 9.13%). We noticed a difference in the distribution pattern of FLAG-IP2 in the double transgenic cells, which was more punctate when co-expressed with LeishIF4E-4 as compared to LeishIF4E-1 and LeishIF4E-3. This variation could result from the overexpression of the different cap-binding proteins. The data obtained in the co-staining experiments using confocal microscopy correlates well with the data obtained from other approaches.

The association between Leish4E-IP2 and the different LeishIF4Es was also examined using yeast two-hybrid (Y2H) assays, as well as co-precipitation experiments, that monitor direct interactions between recombinant proteins. The results shown in Supplementary Figure S4 confirmed the individual interactions between Leish4E-IP2 and LeishIF4E-1, as well as with LeishIF4E-3. No interaction was observed between Leish4E-IP2 and LeishIF4E-4 in the Y2H assay. Despite the association of LeishIF4E-4 with Leish4E-IP2 shown in the pull down assays (Figure 1, Supplementary Figure S2 and Table S4), we were unable to show a direct interaction between those two proteins using a Y2H assays (Supplementary Figure S4). Assays designed to highlight direct interactions were also performed, using recombinant proteins. GST-tagged Leish4E-IP2 and His-tagged LeishIF4E-1 or LeishIF4E-3, were cloned and expressed in *Escherichia coli* (Supplementary Figure S5A). The direct interactions between recombinant GST-Leish4E-IP2 and the His-tagged LeishIF4E-1 and LeishIF4E-3 were confirmed *in vitro*, using co-precipitation assays. Supplementary Figure S5B&C shows that Leish4E-IP2 interacts with LeishIF4E-1, and to a much lesser extent with LeishIF4E-3. The negative control using the GST tag alone, did not interact with LeishIF4E-1 and LeishIF4E-3, indicating that these interactions are specific to Leish4E-IP2. The difference between the binding of Leish4E-IP2 to LeishIF4E-1 and LeishIF4E-3 is also apparent from the Y2H assays in Supplementary Figure S4. Furthermore, as in the Y2H assays, we also could not demonstrate a direct interaction between Leish4E-IP2 and LeishIF4E-4 using the recombinant protein assay (data not shown). It is therefore possible that the expected interaction is indirect, or that it requires specific post-translational modifications that occur only within the parasite cells.

We further investigated the proteomic content of the Leish4E-IP2 associated proteins from cell extracts of *L. amazonensis* that expressed the SBP-tagged Leish4E-IP2. The extracts were affinity-purified over streptavidin beads and the content of the eluted fractions was determined by LC-MS/MS. A control analysis was performed on cells expressing SBP-tagged luciferase. The experimental and control analyses were each performed as three independent repeats. The proteome associated with Leish4E-IP2-SBP was compared with that of Luciferase-SBP, to identify proteins that were relatively enriched in Leish4E-IP2-SBP proteome, using a threshold of at least three-fold (log_2_ > 1.6), with *P* < 0.05. The statistical analysis was carried out by the Perseus software platform (38). Supplementary Figure S6A and Table S4 describe the manually categorized groups of proteins enriched in Leish4E-IP2, as compared to Luciferase-SBP. Leish4E-IP2 associates with proteins involved in RNA binding and metabolism, translation, proteasome, signaling, transport and general metabolism. The diverse categories of proteins associated with Leish4E-IP2.

Leish4E-IP2 associated proteins were also subjected to Gene Ontology (GO) enrichment analysis through the TryTripDB platform, based on their cellular components. Supplementary Figure S6B highlights the major categories of the protein groups, each containing at least seven proteins that were enriched by over 2.5-fold. In line with the manually categorized proteins, the GO enrichment analysis identified proteins related to the proteostasis, i.e. those that associated with translation and proteolysis. The translation-related proteins were derived mainly from the LeishIF4F and LeishIF3 complexes. Other enriched groups include ribonucleoprotein granules, which contain many ribosomal proteins. Similar to the manual categorization, the GO enrichment analysis also highlighted proteins of metabolic pathways, such as the Proton-transporting two-sector ATPase complex. The physiological meaning of the large repertoire of proteins that are part of the Leish4E-IP2 interactome is still not clear to us. We also cannot exclude the possibility that these proteins are associated with Leish4E-IP2 indirectly.

### Expression of LeishIF4E-1 and LeishIF4E-4 along the growth curve

**Figure 2.**
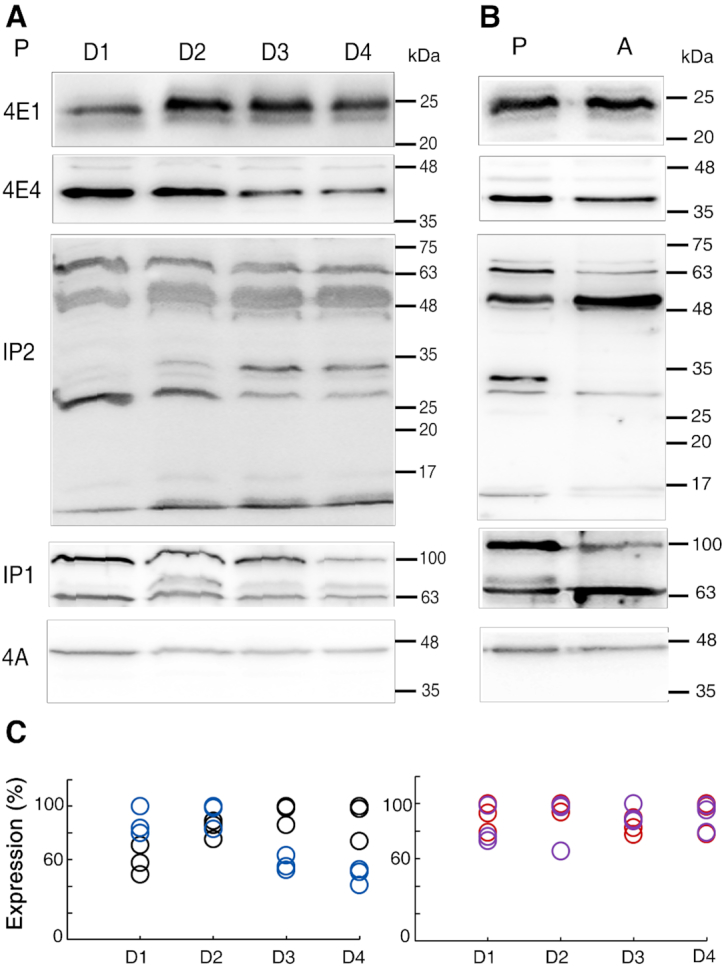
Steady-state expression of LeishIF4E-1, LeishIF4E-4, Leish4E-IP1, Leish4E-IP2 along the growth curve of promastigotes and axenic amastigotes. (**A**) Aliquots were collected daily (D1–D4: days 1–4) from *L. amazonensis* promastigotes and samples containing equal protein loads of cell lysates were resolved by SDS-PAGE. The gels were subjected to western blot analysis with antibodies directed against LeishIF4E-1 (4E1), LeishIF4E-4 (4E4), Leish4E-IP1 (IP1) and Leish4E-IP2 (IP2). LeishIF4A1(4A) was used as a loading control. The multiple bands observed for Leish4E-IP1 and Leish4E-IP2 could suggest that they are susceptible to break down. (**B**) As described in A, except with aliquots obtained from promastigote extracts from a day 2 culture and axenic amastigotes extracts (day 4 following differentiation) separated by SDS-PAGE. (**C**) Dot plot represents densitometry analysis (based on three replicate experiments) of the steady-state expression of LeishIF4E-1 (black circles in the left graph), LeishIF4E-4 (blue circles in the left graph), Leish4E-IP1 (red circles in the right graph) and Leish4E-IP2 (purple circles in the right graph).

The mouse eIF4E binds the m^7^GTP cap-analog with a *K*_ass_ higher by ∼500-fold than the *K*_ass_ values measured for LeishIF4Es 1–4. However, the different LeishIF4Es vary with respect to their binding affinities to different cap analogs. Thus, LeishIF4E-1 and LeishIF4E-4 were both reported to bind m^7^GTP better than LeishIF4E-2 and LeishIF4E-3 (18), although their differential roles during translation initiation remained unclear, especially during the discrete life stages of the parasites. To characterize whether they follow unique expression patterns, we monitored their steady state level along the growth curve of promastigotes, as well as in axenic amastigotes. We also examined whether the expression pattern of Leish4E-IP1 and Leish4E-IP2 changed to evaluate whether they showed some correlation with the expression profile of LeishIF4Es. Early log phase cells (10^6^ cells/ml) were seeded and allowed to grow until they reached stationary phase (2.3 × 10^7^ cells/ml, day 4). Aliquots were collected daily for further analysis by western blots, using antibodies directed against each of the tested proteins. LeishIF4A-1 served as a loading control. Expression levels were quantified by densitometry analysis, and the results were normalized to LeishIF4A-1. Figure 2A shows that the steady-state expression of LeishIF4E-1 was relatively low in early-log cells and increased towards mid- and late-log-stages, whereas expression of LeishIF4E-4 was high in early- and mid-log-phase cells, and decreased after that. This profile was verified by the densitometry analysis (Figure 2C). Expression of Leish4E-IP1 and Leish4E-IP2 appeared to be unchanged and steady along the growth curve of promastigotes, although their migration pattern was intriguing, most probably due to proteolytic cleavage. However, since Leish4E-IP2 appears to be susceptible to proteolytic cleavage, this limits our ability to provide exact quantifications of this protein. Supplementary Figure S7 shows the induction of recombinant Leish4E-IP2 that served for the antibody preparation. When checked with *Leishmania* lysates, the antibodies highlighted several protein bands, suggesting the potential breakdown of Leish4E-IP2. The migration profile observed for the endogenous Leish4E-IP2 was also unusual, since it generated a band that migrated above the 63 kDa marker, higher than expected. This slow migration is speculated to be due to the presence of unstructured modules in the protein (39). The antibodies also interacted with smaller bands (Figure 2A and B). This suggested that Leish4E-IP2 could be susceptible to proteolytic cleavage. The different bands were therefore examined and verified, as shown in Supplementary Figure S8 and Table S5. Cell extracts prepared from transgenic parasites expressing SBP-tagged Leish4E-IP2 were affinity purified over streptavidin-Sepharose beads following elution with biotin. The eluted fraction was separated over 12% SDS-PAGE in parallel lanes. One lane was subjected to western analysis, and its parallel lane served for verification of the proteins extracted from the related band regions, by mass spectrometry. This analysis, shown in Supplementary Figure S8 and Table S5, verified that all the bands that interacted with antibodies against Leish4E-IP2 and antibodies against SBP, contained peptides derived from Leish4E-IP2. The larger band (>63 kDa) was also shown to contain a Leish4E-IP2-derived peptide. Fragments of similar sizes were observed in parasite extracts derived from wild type cells and from transgenic lines that overexpress Leish4E-IP2 (Supplementary Figure S9). Overexpression of the transgenes was stronger in cells transfected with SBP-tagged LeishIF4E-1, LeishIF4E-4 and Leish4E-IP1, whereas expression of transgenic Leish4E-IP2-SBP appeared to be weaker, although such a pattern could be due to the strong cleavage of the protein. The high susceptibility to proteolytic cleavage was also observed for Leish4E-IP1 and its *T. brucei* ortholog, Tb4E-IP1, which breaks down to smaller discrete polypeptides (40).

### Leish4E-IP2 is hardly found in fractions eluted from m^7^GTP-agarose

**Figure 3.**
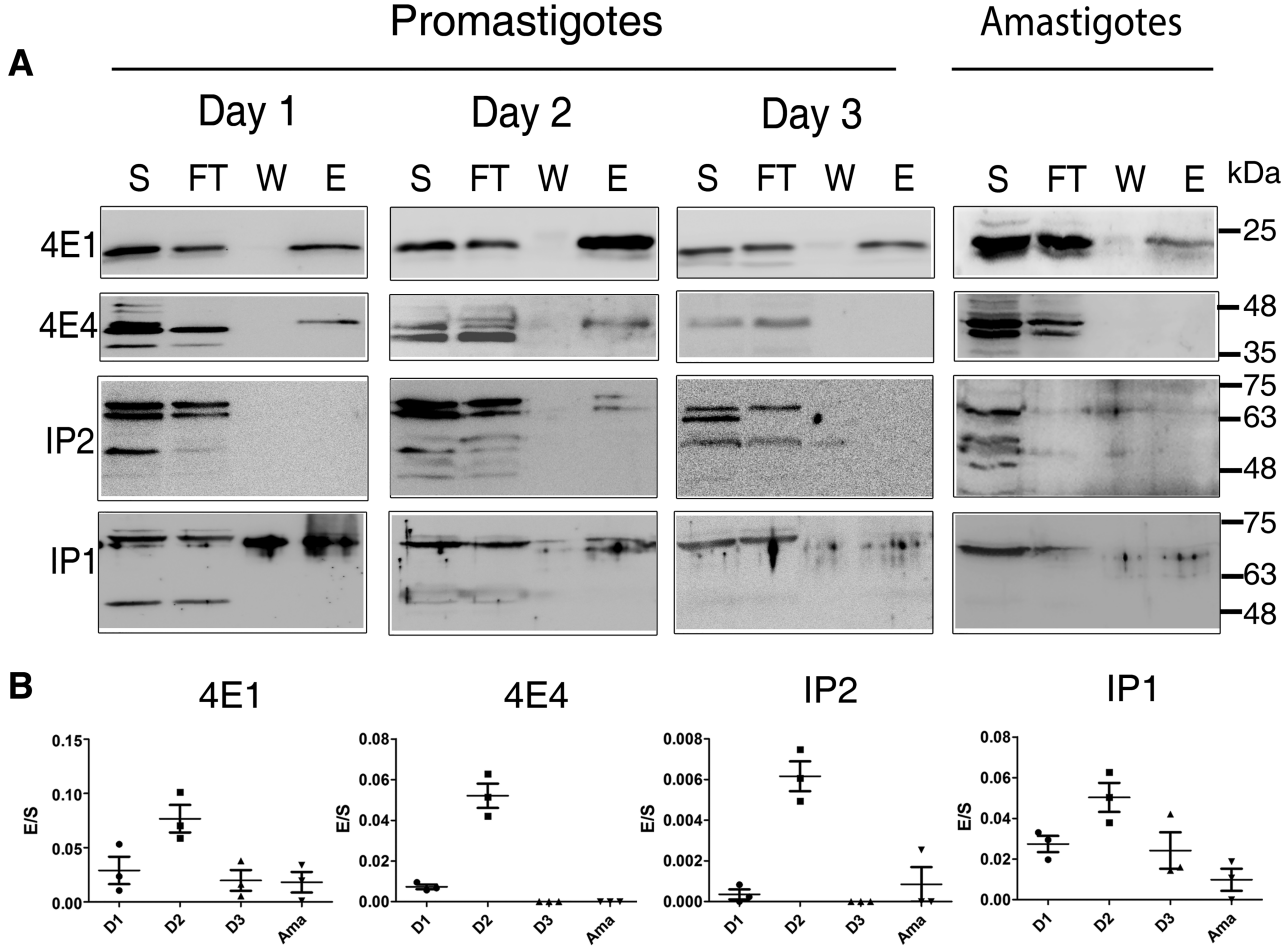
m^7^GTP pulldown assays with wild-type L. amazonensis along the growth curve of promastigotes and axenic amastigotes. (**A**) Cell extracts (from 10^9^ cells) obtained from different growth stages of promastigotes and axenic amastigotes (Amastigotes) were incubated with m^7^GTP-agarose beads. The beads were washed and further eluted with free m^7^GTP. Aliquots of the soluble extract (S, 5%), flow-through (FT, 5%), wash (W, 50%) and the eluted fraction (E, 50%) were separated by SDS-PAGE. Blots were analyzed using specific antibodies directed against LeishIF4E-1 (4E1), LeishIF4E-4 (4E4), Leish4E-IP1 (IP1) and Leish4E-IP2 (IP2). (**B**) The dot plots represent the ratio between the eluted proteins and their original total loads (E/S) obtained from three independent experiments, for each protein. The data are represented as the median of at least three independent experiments. Error bars indicate the standard deviations of the mean.

In addition to the changes reported for their steady-state expression, we measured the cap-binding activities of LeishIF4E-1 and LeishIF4E-4 along the growth curve of promastigotes, and in amastigotes. In the absence of a resin with immobilized cap-4, we used beads with m^7^GTP bound to agarose. Both proteins were previously shown to bind both m^7^GTP and cap-4 analogs (18), but their different affinities should be recognized. Early log phase cells (10^6^ cells/ml) were seeded and allowed to grow until they reached stationary phase (2.3 × 10^7^ cells/ml, day 4). The cells were analyzed by affinity purification over m^7^GTP-agarose beads at different stages of growth curve. The beads were eluted by free m^7^GTP, and aliquots from the supernatant, flow through, wash, and the eluted fractions were resolved by SDS-PAGE and further subjected to Western Blot analysis with antibodies directed against LeishIF4E-1, LeishIF4E-4, Leish4E-IP1 and Leish4E-IP2 (Figure 3A). We did not analyze the presence of LeishIF4E-3, as it is known to be a weak cap-binding protein (16,18). The blots were subjected to densitometry analysis, and the results are shown in Figure 3B. The cap-binding activity of LeishIF4E-4 was highest in the mid-log-phase of the promastigote stage (day 2) and was hardly detected in late log cells (day 3), as well as in the amastigote life-forms. LeishIF4E-1, however, did bind to m^7^GTP at all different points of the growth curve, with the highest binding observed in mid-log phase. This altered profile of cap-binding activity could suggest that the two proteins are assigned different tasks along the growth curve, and that they could be subject to a fine-tuning of translation regulation that also affects their expression level.

As shown in Figure 3, Leish4E-IP1 was eluted from the m^7^GTP-agarose column at the early growth phases of promastigotes, and reduced towards stationary phase and in axenic amastigotes. Unlike Leish4E-IP1, Leish4E-IP2 was not eluted from the m^7^GTP-agarose beads at most time point along the promastigote growth curve (except for a low elution in day 2), or in amastigotes, suggesting that it is excluded from binding LeishIF4E-1 or LeishIF4E-4 when these are bound to the cap.

### Overexpression of Leish4E-IP2 reduces the cap-binding activity of LeishIF4E-1, LeishIF4E-3 and LeishIF4E-4 in promastigotes and inhibit translation

**Figure 4.**
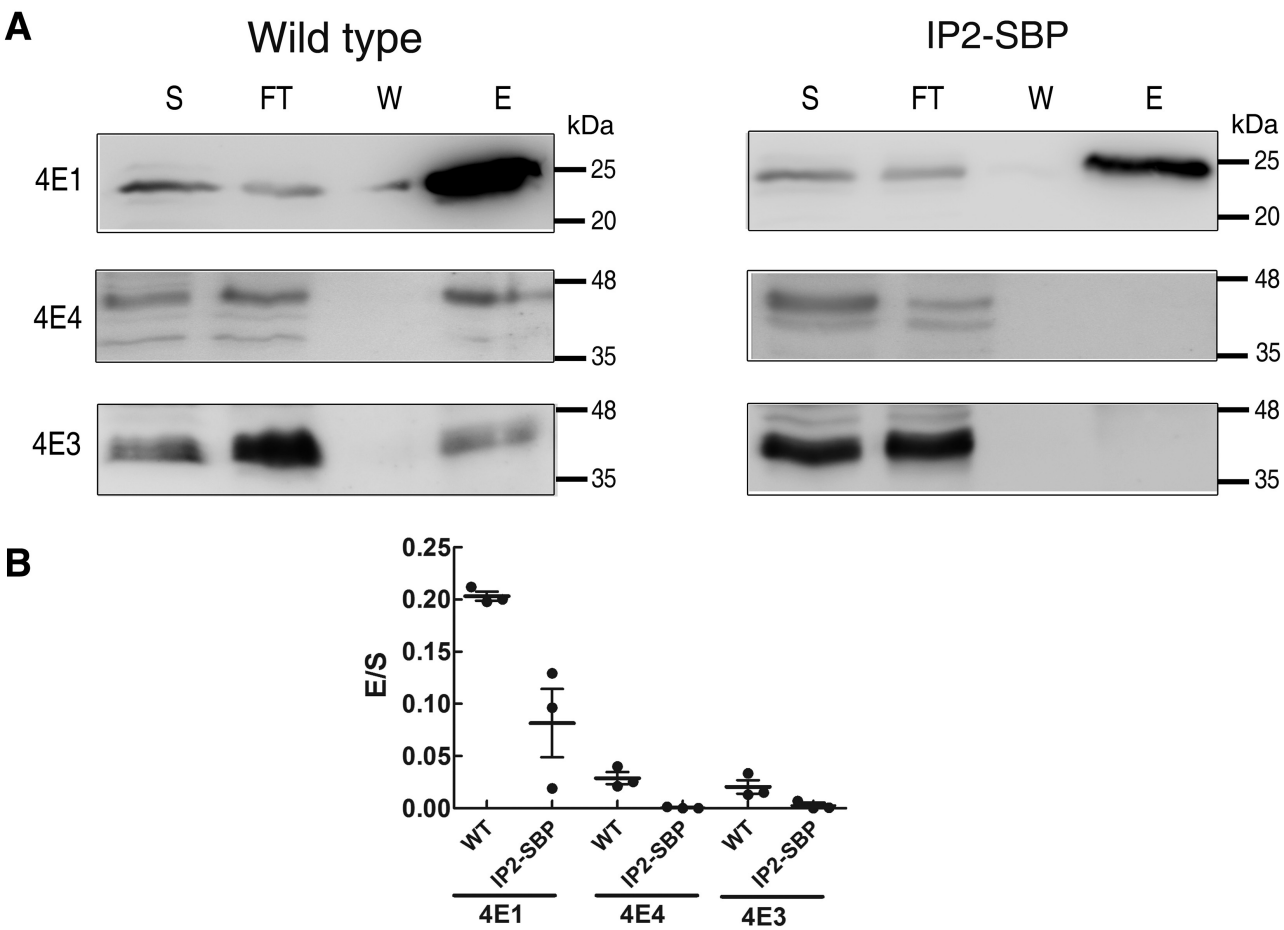
Co-purification of cap-binding proteins over m^7^GTP-agarose from day 2 of wild type L. amazonensis cells and in Leish4E-IP2 over-expressing cells. (**A**) Extracts obtained from wild type cells (left panel) and from transgenic cells that overexpress Leish4E-IP2 (right panel) were lysed and incubated with m^7^GTP-agarose beads. The beads were washed and further eluted with free m^7^GTP. Aliquots of the soluble extract (S, 5%), the flow-through fraction (FT, 5%), wash (W, 50%) and the eluted fraction (E, 50%) were separated by SDS–PAGE and further subjected to western blot analysis. The blots were analyzed using specific antibodies directed against LeishIF4E-1, LeishIF4E-4 and LeishIF4E-3. (**B**) The dot plots represent the ratio between the eluted proteins and their corresponding total loads in each experimental group (E/S). The ratios of eluted proteins in wild type (black) and IP2-SBP (grey) cells are represented by the black and gray columns, respectively, and calculated from three independent repeats. Error bars indicate the standard deviations of the mean.

Following our earlier reported observation that Leish4E-IP1 regulates the cap-binding activity of LeishIF4E-1 (36), we further asked whether over-expression of Leish4E-IP2 affected the cap-binding activity of the different LeishIF4Es. This was examined in transgenic cells that expressed Leish4E-IP2-SBP and compared to wild type *L. amazonensis* promastigotes. Extracts from both cell lines were loaded on an m^7^GTP-agarose resin. The beads were washed and eluted with free m^7^GTP. Aliquots from the different fractions were examined by SDS-PAGE and followed by western blot analysis. Figure 4 shows that over-expression of Leish4E-IP2 in transgenic *L. amazonensis* cells led to a decrease of the cap-binding activity of LeishIF4E-1 (to 50%), LeishIF4E-3 (to 85%) and LeishIF4E-4 (95%). Thus, Leish4E-IP2 not only binds the different cap-binding proteins but also reduces their cap-binding activity. Given that Leish4E-IP2 did not associate with cap-binding protein complexes (Figure 3) and that its over-expression indeed reduced the cap-binding activity of LeishIF4E-1, LeishIF4E-3 and LeishIF4E-4, as compared to wild type cells, we examined whether this inhibition also affected global translation levels.

**Figure 5.**
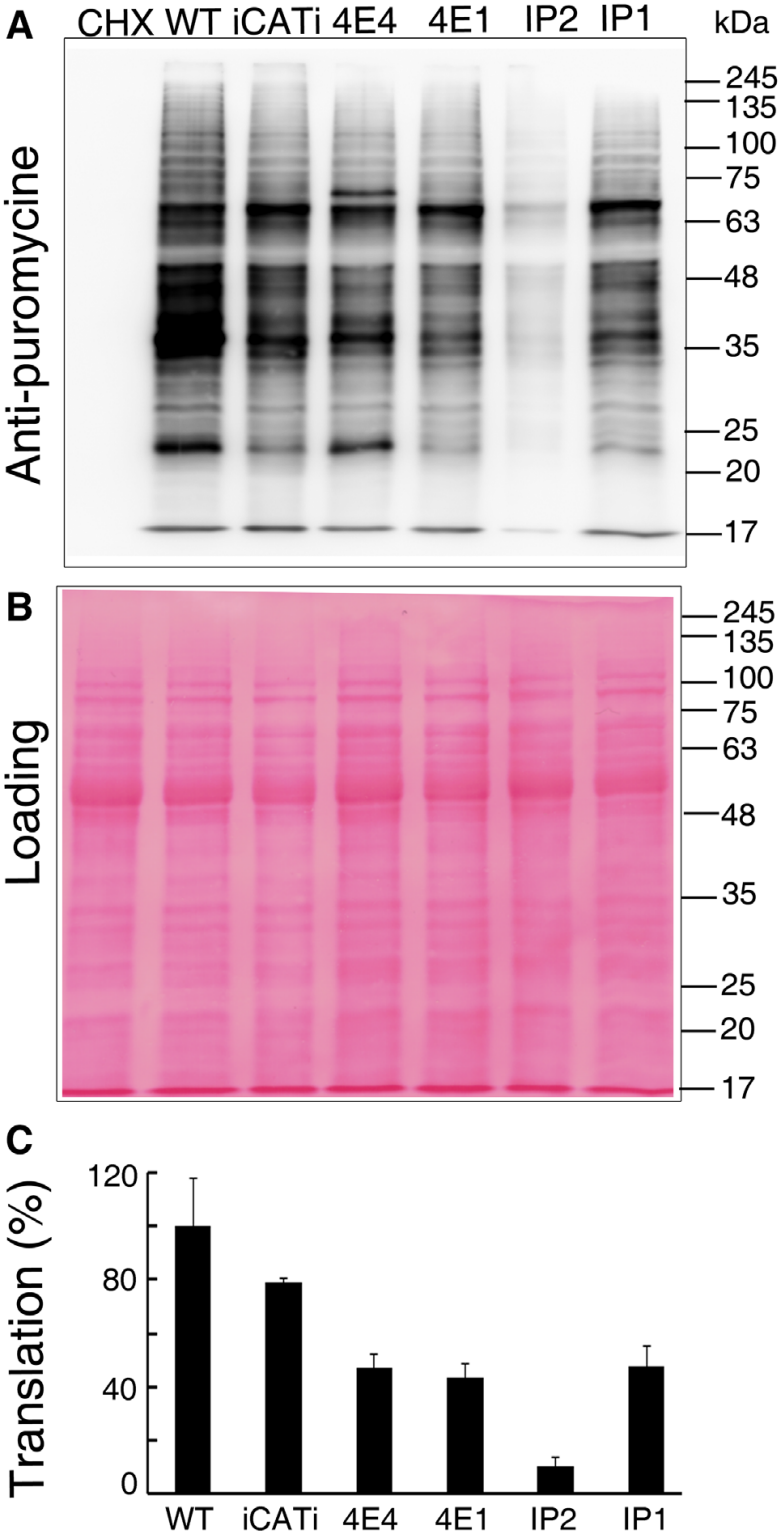
Over-expression of Leish4E-IP2 reduces translation. (**A**) The translation was demonstrated by the SUnSET translation assay that monitors the incorporation of puromycin into actively translating ribosomes. The experiment was performed on mid-log wild type *L. amazonensis* cells, along with cells that over-express LeishIF4E-1 (4E1), LeishIF4E-4 (4E4), Leish4E-IP2 (IP2) and Leish4E-IP1 (IP1). A control cell line that expresses the CAT reporter gene (also under control of the *HSP83* intergenic regions) was included. Cells incubated with cycloheximide (CHX) served as a negative control for complete translation arrest. Whole-cell extracts were separated by SDS-PAGE and subjected to western blot analysis, using specific antibodies directed against puromycin. (**B**) Ponceau staining of the blot demonstrating equal protein loads. (**C**) Densitometry analysis of the puromycin incorporation in the different transgenic cell lines, as compared to wild type cells (considered as 100%).

Accordingly, we performed a SUnSET assay, which is based on the incorporation of puromycin, a tRNA analog, into the A-site of translating ribosomes (35). Puromycin incorporation in growing polypeptide chains was monitored in cell extracts and resolved by SDS-PAGE followed by western blot analysis, using anti-puromycin antibodies. This assay was performed on active mid-log cells (day 2) over-expressing LeishIF4E-1, LeishIF4E-4, Leish4E-IP1 or Leish4E-IP2, because translation activity peaked at that time point (Supplementary Figure S10). Global translation in these cell lines was compared with that measured in wild type cells, along with a control of a transgenic cell line that over-expressed the chloramphenicol acetyltransferase reporter gene (CAT), under control of the HSP83 intergenic regions. Figure 5 shows that over-expressing Leish4E-IP2 reduced the translation level to 10% of that measured in the wild type cells. Translation levels in cells over-expressing other proteins, such as the chloramphenicol acetyl transferase (CAT) reporter gene [as an over-expression control (41)], LeishIF4E-4, LeishIF4E-1 and Leish4E-IP1 were 78.7%, 46.9%, 43.7%, 47.7% of wild type cells (100%), respectively. Thus, over-expression of foreign or endogenous transgenic proteins (as shown in Supplementary Figure S9) had a negative effect on global translation, possibly by competing for translation factors and ribosomes (Figure 5). However, despite this reduction, overexpression of Leish4E-IP2 had a much stronger inhibitory effect, suggesting it could be related to a specific function of Leish4E-IP2. Further, we show that parallel to the translation inhibition observed in cells that overexpress Leish4E-IP2, growth of these cells was impaired as compared to other cell lines and wild type cells (Supplementary Figure S11). Our data, therefore, suggest that Leish4E-IP2 is an important regulatory protein with an impact on global translation level.

### Recombinant Leish4E-IP2_1-134_ inhibits the cap-binding activity of LeishIF4E-1 and -3

**Figure 6.**
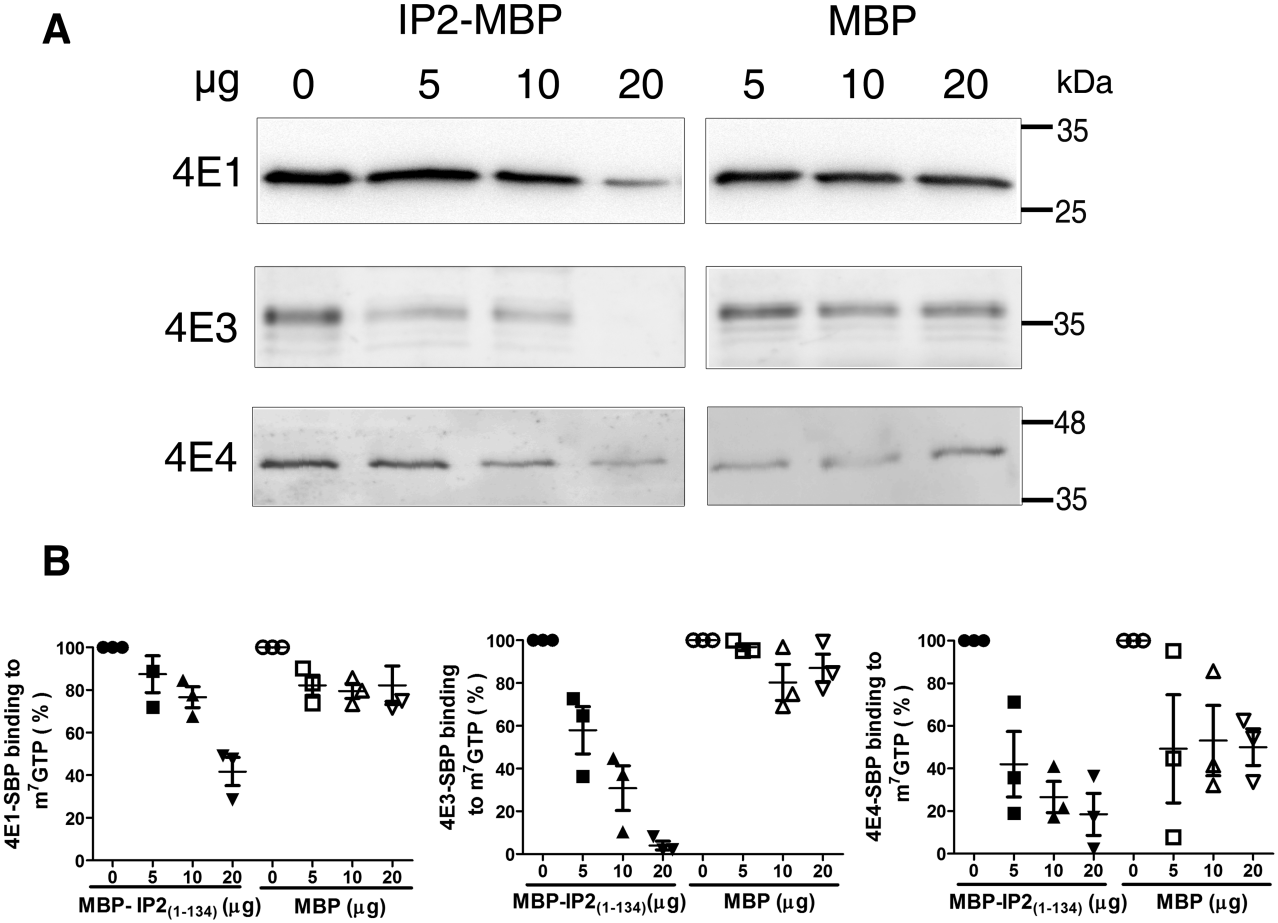
Recombinant Leish4E-IP2_1–134_ controls m^7^GTP cap-binding activity of various LeishIF4Es. (**A**) Cell extracts from Streptavidin-binding peptide (SBP)-tagged LeishIF4E-1, LeishIF4E-3 and LeishIF4E-4 were affinity-purified over an m^7^GTP agarose beads, in the presence or absence of an increasing amount of purified recombinant MBP tagged Leish4E-IP2_1–134_ (that includes the Y(X)_4_LΦ motif), or MBP protein alone as a control. Proteins that remained bound to m^7^GTP after several washes were boiled in sample buffer and were separated on SDS-PAGE and analyzed by western blot using an anti-SBP antibody, detecting LeishIF4Es. (**B**) Densitometry analysis of the blot shown in the upper panel. Quantitative reduction by MBP tagged Leish4E-IP2_1-134_ or MBP on the binding of LeishIF4E-1, LeishIF4E-3 and LeishIF4E-4 to the m^7^GTP beads is shown as a percentage value when compared to SBP-LeishIF4Es in the absence of Leish4E-IP2_1–134_ (0 μg, set as 100% signal). The data are represented as the mean of at least three independent experiments. Error bars indicate the standard error of the mean.

We further validated our preliminary observation that overexpression of Leish4E-IP2 reduces the cap-binding activity of different LeishIF4Es using the recombinant version of Leish4E-IP2, represented by its N-terminal region 1–134 that contains the Y(X_4_)LL motif (the Leish4E-IP2_1–134_ fragment was chosen to prevent us from working with degradation products of Leish4E-IP2; Figure 6). We induced the expression of recombinant MBP-Leish4E-IP2_1–134_ (Supplementary Figure S12) and monitored its effect on the cap-binding activities of LeishIF4Es in a dose-dependent manner. *L. amazonensis* cells overexpressing SBP-tagged LeishIF4E-1, LeishIF4E-3 and LeishIF4E-4 were incubated with different concentrations of recombinant and purified MBP-4E-IP2_1–134_ (0, 5, 10, 20 μg), or with purified MBP alone (0, 5, 10, 20 μg) as control. Following this incubation, the mixtures were further incubated with m^7^GTP-agarose beads. The beads were washed three times and eluted by the addition of SDS-Laemmli's buffer and boiling for 5 min. The eluted proteins were subjected to Western Blot analysis and the ratio between the eluted fraction of the LeishIF4Es and their amount in the total load was determined. Figure 6A shows that Leish4E-IP2_1–134_ reduces the cap-binding activity of LeishIF4E-1 in a dose-dependent manner, starting in the presence of 5 ug (13%) but with the dominant effect in the presence of 20 μg of Leish4E-IP2 (42%). The control MBP alone did not affect the binding of LeishIF4E-1 to the m^7^GTP cap. The inhibitory effect of Leish4E-IP2 was even more prominent with LeishIF4E-3, since Leish4E-IP2_1–134_ reduced the binding of LeishIF4E-3 to the cap already in the presence of 5μg Leish4E-IP2_1–134_ (60%) and addition of 20 μg Leish4E-IP2_1-134_ inhibited the cap-binding activity of LeishIF4E-3 almost completely (96%; Figure 6B). Unlike our observations with LeishIF4E-1 and −3, we could not establish this direct effect of Leish4E-IP2_1–134_ on the cap-binding activity of LeishIF4E-4, due to the non-specific binding of MBP to LeishIF4E-4.

## DISCUSSION

Given that multiple LeishIF4E paralogs are expressed in *Leishmania*, we expect to find a regulatory network of proteins that modulates their expression and activities along the parasite life cycle. In this study, we report on Leish4E-IP2, a novel IF4E-interacting protein in *Leishmania*. The protein was initially identified in an *in vivo* pull-down experiment using a transgenic SBP-tagged LeishIF4E-1. Further analysis showed that in addition to its ability to bind LeishIF4E-1, Leish4E-IP2 could also bind other LeishIF4Es, including LeishIF4E-3 and LeishIF4E-4. These three paralogs were selected as they previously showed differential activities under altered environmental conditions (20,31). LeishIF4E-1 and LeishIF4E-4 are known to possess an efficient cap-binding activity and are therefore assumed to be involved in translation. We found that Leish4E-IP2 did not elute from the m^7^GTP-agarose column, and its over-expression reduced the cap-binding activity of the three tested SBP-tagged endogenous LeishIF4Es and decreased global translation in promastigotes. Furthermore, the purified recombinant Leish4E-IP2 (1–134) fragment reduced the cap-binding activity of LeishIF4E-1 and LeishIF4E-3. Based on the function of Leish4E-IP2 on reducing the cap-binding activity of LeishIF4Es and inhibiting cellular translation, we suggest that Leish4E-IP2 functions as a translation repressor.

Most eIF4E-binding proteins contain the consensus motif Y(X)_4_LΦ, which was shown to directly interact with their target eIF4E (4,6,36,42,43). However, exceptions for eIF4E-interacting proteins that lack this element are also known (15). Proteins that are part of a given complex can be identified by pull down experiments with a tagged trans-gene encoded protein. Using this approach, we identified Leish4E-IP2, a non-annotated hypothetical protein that contains the consensus binding motif Y(X)_4_LΦ. In this study, we showed that Leish4E-IP2 associates with a broader range of LeishIF4Es, including LeishIF4E-1, LeishIF4E-3 and LeishIF4E-4. These associations were shown by various methods, including pull down assays with tagged LeishIF4Es along with reciprocal assays, using SBP-tagged Leish4E-IP2. Mass spectrometry analysis of Leish4E-IP2 pull down samples not only confirmed this association but also identified an additional cap-binding paralog, LeishIF4E-5, that co-precipitated with Leish4E-IP2. The co-staining of Leish4E-IP2 with the different LeishIF4Es *in vivo* was further demonstrated in confocal microscopy experiments. The Leish4E-IP2, along with LeishIF4Es aggregated within the cytoplasm, as seen by their punctate staining pattern. This could suggest that Leish4E-IP2 foci could serve as storage sites for inactive LeishIF4Es. However, further investigation is required to understand the nature and function of these cytosolic aggregates.

The direct interactions between Leish4E-IP2 and LeishIF4E-1 or LeishIF4E-3, were established using *in-vitro* assays with recombinant proteins, and yeast two-hybrid assays. This approach verified the ability of Leish4E-IP2 to directly interact with LeishIF4E-1 and LeishIF4E-3 (to a lower extent), but not with LeishIF4E-4. However, LeishIF4E-4 showed a clear association with Leish4E-IP2 in reciprocal pull-down assays from transgenic parasite cell lines, suggesting that a direct interaction between the two proteins could require additional proteins to stabilize the complex, which may not be present in bacteria or yeast. We therefore show that Leish4E-IP2 associates with LeishIF4E-4, but we could not establish the direct interaction between them.

Unraveling the roles of the multiple LeishIF4E paralogs is challenging. They vary in their relative expression and cap-binding activity, not only between promastigotes and amastigotes, but also along the growth curve of promastigotes. We show here that the relative expression of LeishIF4E-4 is high in early and mid-log cells and reduces thereafter, whereas the relative steady-state expression of LeishIF4E-1 increases in mid log cells, stays high in their late log and towards the stationary phases of parasite growth. We expect that the cellular machinery that is responsible for differential gene expression regulates their altered expression profiles. Leish4E-IP2, appeared to express uniformly along the growth curve of promastigotes and in amastigotes. However, since Leish4E-IP2 is susceptible to degradation, we could not determine whether and how these degradation products affected the interaction of Leish4E-IP2 with its partners.

LeishIF4E-1 and LeishIF4E-4 also vary in their cap-binding activities along the growth curve of promastigotes. LeishIF4E-4 shows its highest binding activity in early and mid-log phase, whereas the LeishIF4E-1 binding activity is low in early log cells, increases in mid-log cultures and is maintained also in later stages of growth, when LeishIF4E-4 does not bind the cap. Changes are also observed between promastigotes and amastigotes (20), since only LeishIF4E-1 continues to bind the cap-structure in amastigotes. However, in all life stages, Leish4E-IP2 does not associate with the cap-binding complex, except for a minor fraction of this protein that co-purified over m^7^GTP-agarose in mid log promastigotes (day 2 cultures). Overall, the function of cap-binding proteins appears to require a complex network of Leish4E-interacting proteins that can control their cap-binding activities. One example for a protein that regulates LeishIF4E activity is Leish4E-IP1, a 4E-interacting protein that specifically binds to LeishIF4E-1. The structural basis for its interaction with LeishIF4E-1 was recently reported (36). However, the role of Leish4E-IP1 can provide only a partial explanation of how the IF4E-regulatory network functions, because it binds exclusively to LeishIF4E-1 and not to other LeishIF4Es. Thus, we anticipated that additional Leish4E-IP proteins that bind specifically to other LeishIF4Es are part of the network that regulates translation, Leish4E-IP2 being such a candidate. Identification of Leish4E-IP2 renders further analysis of its mode of interaction with other LeishIF4Es.

Studies on the *T. brucei* ortholog of Leish4E-IP1, Tb4E-IP1, based on its tethering to the 3′ UTR of the CAT reporter transcript, repressed the reporter gene translation. Furthermore, Tb4E-IP1 also promoted mRNA degradation, even in the absence of any known motifs typical of mRNA binding (40). A tethering assay revealed that the tethered TbIF4E-1 alone did not suppress expression of the reporter gene. However, when Tb4E-IP1 was tethered along with TbIF4E-1, expression of the reporter gene reduced. Tethering of Tb4E-IP1 alone also reduced this expression, indicating that Tb4E-IP1 is a translation repressor (40). The *T. brucei* ortholog of Leish4E-IP2 was not found in the final list of the genome-wide tethering screen (44), therefore we could not assign any role for this protein in terms of translation regulation.

The function of Leish4E-IP2 association with LeishIF4Es was initially investigated by their co-purification with SBP-tagged LeishIF4Es over streptavidin-Sepharose columns. Their effect on the cap-binding activities of the different LeishIF4Es was further examined by affinity-purification assays over m^7^GTP-agarose. These excluded their ability to assemble over the cap structure. A further support to this observation was obtained from *in vitro* experiment using a functional assay, in which the recombinant Leish4E-IP2_1–134_ directly inhibited the cap-binding activities of LeishIF4E-1 and LeishIF4E-3, in a dose-dependent manner. In accordance, we found that overexpression of Leish4E-IP2 inhibited global translation.

Based on our results, we propose that Leish4E-IP2 is a general inhibitor of cap-binding activity and hence of translation. However, the mechanism of action of Leish4E-IP2 could be further understood by structural studies. In Opisthokonts, assembly of the cap-binding translation initiation complex can be globally regulated by the 4E-binding protein (4E-BP). 4E-BP senses cell signals through the kinase activity of mTOR. The phosphorylation of 4E-BP reduces its affinity to eIF4E, which activates translation initiation (45). An additional 4E-binding protein that regulates translation of specific mRNAs is the 4E-transporter (4E-T). 4E-T is a nucleocytoplasmic shuttling protein which possesses the conserved 4E-binding motif Y(X_4_)LΦ, its binding to eIF4E reduces global translation (46,47). 4E-T homologs in *Drosophila* and *C. elegans* (Cup and Spn-2, respectively) act during early development to repress translation of *oskar, nanos* in *Drosophila* and *katanin* mRNA in *C. elegans* (48,49). 4E-T also binds RNA-binding proteins, particularly DDX-6 that is involved in miRNA-dependent translation repression and P-body assembly (46,47). Another 4E-binding protein involved in translation repression is GIGYF2, which forms a complex with 4E-HP, a cap-binding repressor, to halt translation of a subset of mRNAs during embryonic development in mammals (50). The various 4E-binding proteins are thus all involved in either global or gene-specific translation repression. In the case of Leish4E-IP2, even though it does not show any sequence homology with these proteins, it appears to serve a parallel function in down-regulating translation in *Leishmania*. There are no 4E-BP homologs in the *Leishmania* or trypanosome genome databases, but other regulatory proteins could replace them in a manner suited to the requirements of the parasite. The broader affinity of Leish4E-IP2 to different LeishIF4Es can be explained if the latter function as transcript-specific translation factors. In such case, the ability of Leish4E-IP2 to associate with different LeishIF4Es could broaden its repressive activity. Transcript-specific translation could very well be supported by the involvement of RNA binding proteins that associate with the different LeishIF4Es. To date, we reported on transcript-specific association of discrete mRNAs with LeishIF4E-3 (30).

Given the complex translation system of *Leishmania*, the existence of a network of regulatory Leish4E-interacting proteins is required to control the cap-binding activity and translation. Leish4E-IP2 is one such regulatory protein that has an impact on global translation. We assume that additional LeishIF4E interacting proteins will be identified in the future and it should be interesting to examine whether they function independently or as part of a regulated network.

## Supplementary Material

gkaa173_Supplemental_Files
