# Supplementary material for: A newly identified Leishmania IF4E-interacting protein, Leish4E-IP2, modulates the activity of cap-binding protein paralogs
Source: Nucleic Acids Res. 2020 Mar 30;48(8):4405–17. doi: 10.1093/nar/gkaa173 (PMC7192595; doi:10.1093/nar/gkaa173)
Supplement: gkaa173_Supplemental_Files [file gkaa173_supplemental_files.zip › Supplemental materials - Final_NoMarks.pdf]

SUPPLEMENTAL DATA

MATERIALS AND METHODS

Sequence alignment of the novel Leish4E-interacting protein 2 (Leish4E-IP2) in *Leishmania*
The open reading frames of Leish4E-IP2 orthologs derived from different *Leishmania* and *Trypanosoma* species were aligned using Jalview (2.10.5). The sequences were derived from *L. amazonensis* (L. ama, LAMA_000698700), *L. major* (L. maj, LmjF33.0380); *L. infantum*, (L. inf, LinJ_330010200)*, T. brucei* (Tb927.10.11000) and *Bodo saltans* strain *Lake Konstanz* (BSAL_75100). The alignment file was saved in a FASTA format. The secondary structure of Leish4E-IP2 was predicted using the online PHYRE^2 server (Protein Homology/analogy Recognition Engine V 2) and saved in a PDB format. The final alignment file showing the predicted secondary structure was developed using the online ESPript 3 tool (1).

Interaction between recombinant LeishIF4E-1 and Leish4E-IP2
We transformed *E. coli* BL21 cells with plasmids allowing the expression of LeishIF4E-1 or LeishIF4E-3 and Leish4E-IP2 as fusion proteins bearing 6X His and glutathione sulfotransferase (GST), respectively (2). Tags were fused at the C-termini of the proteins. We harvested 200 ml of these bacterial cultures, washed with lysis buffer (20 mM Tris-HCl pH 8, 200 mM NaCl, 1 mM EDTA, 5 mM $MgCl_2$, 5% glycerol), and resuspended in the same buffer supplemented with protease inhibitors, benzonase nuclease (Sigma) and 0.1% NP-40. We then disrupted the cells with a French press apparatus and clarified the lysate by centrifugation at 10000g for 1 h. We further clarified the supernatant using 0.4 µM filters. We pre-equilibrated the glutathione resin (50 µl, GeneScript) with 10 volumes of lysis buffer. The lysates of bacterial cells expressing recombinant Leish4E-IP2-GST or GST alone (negative control) were bound to the glutathione resin for 2 h at 4°C with constant shaking. The beads were washed twice with ten volumes of lysis buffer containing 400 mM NaCl and twice with lysis buffer containing 200 mM NaCl. Beads presenting immobilized bait proteins were then incubated with 1 ml of lysate derived from cells expressing His-tagged LeishIF4E1 or LeishIF4E-3 for 2 h at 4°C with constant shaking. The beads were washed with 1 ml cold lysis buffer five times, after which the bound proteins were eluted by boiling the beads with 100 µl SDS sample buffer. Aliquots derived from the supernatant (S, 10%), the flow through (FT, 10%), the wash (W, 25%) and the eluted fractions (E, 25%) were separated over SDS-PAGE, and further subjected to western analysis, using antibodies against LeishIF4E-1 (marked as 4E1) and LeishIF4E-3 and against GST (marked as IP2-GST and GST). A similar control experiment was carried out with cells expressing the GST control alone as a negative control.

Confocal microscopy

We investigated the co-localization of Leish4E-IP2 with different cap-binding proteins *in situ,* using an immuno-histochemical approach. We generated double transgenic cell lines co-expressing FLAG-Leish4E-IP2 along with the individual SBP-tagged cap-binding proteins LeishIF4E-1, LeishIF4E-4, and LeishIF4E-3. We harvested mid-log-phase cells (~10^7 cells/ml), washed with PBS and fixed with 2% paraformaldehyde for 30 min. We then washed the cells with PBS, loaded onto poly-L-lysine-coated slides for 5 min and washed again with PBS. We permeabilized the cells with 0.1% Triton X-100 in PBS, re-washed and blocked with 2% bovine serum albumin (BSA) in PBS for 1 hour at room temperature. We incubated the fixed cells with the relevant primary antibodies [rabbit anti-FLAG (Sigma) for Leish4E-IP2 and mouse monoclonal anti-SBP (Millipore) for LeishIF4Es] for 1 hour at room temperature. We washed the slides and incubated with secondary antibodies fused to different fluorophores [anti-mouse antibodies labeled with Alexa Fluor 488 (green), and anti-rabbit antibodies labeled with DyLight 550 (red)]. We stained nucleic acids with 4′,6-diamidino-2-phenylindole (DAPI, Sigma) (1 µg/ml). Finally, we washed the cells three times with PBS and covered in the presence of anti-bleach mounting solution (DABCO). We examined the slides in an inverted Zeiss LSM 800 spinning disc confocal microscope with Airyscan at a magnification of x63.

Yeast two-hybrid assay

A yeast two-hybrid assay was performed using the GAL4 Two-Hybrid Phagemid Vector Kit (Stratagene). The ORFs of LeishIF-4E1, LeishIF-4E3 and LeishIF-4E4 were cloned into the GAL4–binding domain vector (pBD). The ORFs of Leish4E-IP2 and Leish4E-IP1 were cloned into the GAL4 activation domain vector (pAD). Empty pBD and pAD vectors were used to exclude leakiness of the opposite plasmids. The YRG-2 yeast strain (Mata ura352 his3-200 ade2-101 lys2-801 trp1-901 leu2-3 112 gal4-542 gal80-538 LYS2::UASGAL1-TATA GAL1-HIS3 URA3::UASGAL4 17mers(x3) TATACYC1-lacZ) was co-transfected with the defined pBD and pAD clones and yeast were spotted onto SD-2 (-Trp/-Leu) and SD-3 (-Trp/-Leu/-His) plates, with or without 3-amino-1,2,4-triazole (3-AT, 1 mM) (Sigma). Plasmids pAD-Leish4EIP1 and pBD-Leish4E-1 served as positive controls (3).

LC-MS/MS analysis of Affinity purified SBP-tagged proteins over streptavidin-Sepharose beads

Different cell lines expressing SBP-tagged Leish4E-IP2, LeishIF4E-1 and Luciferase (~10^9 cells) were harvested and washed twice with PBS, once with PRS and lysed with 1% Triton X-100 in PRS+, in a total volume of 1.2 ml over a 5 min period, on ice. The lysates were further centrifuged at 20,000g for 20 min at 4°C. The clarified supernatant was incubated (1.2 ml) with 75 µl streptavidin-Sepharose beads (GE Healthcare) for 2 h and washed three times with PRS+. The final elution was

performed with 5 mM biotin in PRS+. SBP-tagged Leish4E-IP2, LeishIF4E-1 and Luciferase samples after purification over streptavidin beads were subjected to LC-MS/MS analysis.

Verification of Leish4E-IP2 cleavage products observed in western blots

Since the antibodies against Leish4E-IP2 highlighted several protein bands that could indicate on potential breakdown, we analyze the different gel slices obtained from parallel lanes, to verify the presence of Leish4E-IP2 peptides. The migration profile observed for Leish4E-IP2 was intriguing, since it also generated a band that migrated above the 63 kDa marker, higher than expected. The antibodies also interacted with smaller bands. Cell extracts prepared from transgenic parasites expressing SBP-tagged Leish4E-IP2 were affinity purified over streptavidin-Sepharose beads following elution with biotin. The eluted fraction was separated over 12% SDS-PAGE in parallel lanes. One lane was subjected to western analysis, and its parallel lane served for verification of the proteins extracted from the related band regions, by mass spectrometry.

Mass Spectrometry (MS)

Proteins were reduced using 3 mM DTT (60°C for 30 min), followed by modification with 10 mM iodoacetamide in 100 mM ammonium bicarbonate for 30 min at room temperature. This was followed by overnight digestion in 10 mM ammonium bicarbonate in trypsin (Promega) at 37°C. Trypsin-digested peptides were desalted, dried, resuspended in 0.1 % formic acid and resolved by reverse phase chromatography over a 30 min linear gradient with 5% to 35% acetonitrile and 0.1 % formic acid in water, a 15 min gradient with 35% to 95% acetonitrile and 0.1 % formic acid in water and a 15 min gradient at 95% acetonitrile and 0.1 % formic acid in water at a flow rate of 0.15 μl/min. MS was performed using a Q-Exactive Plus Mass Spectrometer (Thermo) in the positive mode set to conduct a repetitively full MS scan followed by high energy collision dissociation of the 10 dominant ions selected from the first MS scan. A mass tolerance of 10 ppm for precursor masses and 20 ppm for fragment ions was set.

Statistical analysis for enriched proteins

Raw mass spectrometric data were analyzed by the MaxQuant software, version 1.5.2.8 (4). The data were searched against the annotated *L. amazonensis* proteins from the TriTrypDB (5). Protein identification was set at less than a 1% false discovery rate. The MaxQuant settings selected were a minimum of 1 razor/unique peptide for identification, a minimum peptide length of six amino acids and a maximum of two mis-cleavages. For protein quantification, summed peptide intensities were used. Missing intensities from the analyses were substituted with values close to baseline only if the values were present in the corresponding analyzed sample. The $\log_2$ of iBAQ intensities (6) were compared between the three biological repeats of each group on the Perseus software platform (7),

using a t-test. The enrichment threshold was set to a $\log_2$ fold change > 1.6 and $p < 0.05$. The annotated proteins were first categorized manually.

Categorization of enriched proteins by the Gene Ontology (GO) Annotation via TriTrypDB

Enriched proteins were classified by the GO Annotation tool in TriTrypDB, based on molecular functions. The threshold for the calculated enrichment of proteins based on their GO terms was set at 2.5 fold, with a $p<0.05$. This threshold eliminated most of the general groups that represented parental GO terms. GO terms for which only a single protein was annotated were filtered out as well. In some cases, GO terms that were included in other functional terms are not shown, leaving only the representative GO term

Expression and purification of Leish4E-IP2$_{1\text{-}134}$-MBP

Recombinant IP2 (1-134)-MBP was expressed in Rosetta strain of *E. coli*. The cells were grown to a density of OD_{600} 0.6 and expression was induced by the addition of 1 mM IPTG at 19°C for 14-16 hr. The cells were harvested, and resuspended in lysis buffer (20 mM Tris-HCl pH 8.0, 200 mM NaCl, 1mM EDTA, protease inhibitor cocktail and 5µg/ml DNaseI). The cells were disrupted using a French Press at 1500 psi, followed by centrifugation at 45,000 rpm (Beckman 70 Ti rotor) and the supernatant was loaded on Amylose resin. The column was equilibrated with 2 Column Volumes (CV) of buffer 1 (20 mM Tris-HCl pH 8.0, 200 mM NaCl, 1mM EDTA). Three washes were carried out with buffer 1 to reduce the non-specific protein binding. Finally, MBP-IP2 eluted with buffer 1 containing 10mM maltose. The sample was dialyzed (three exchanges during 18 h at 1:100 dilutions against Tris-HCl pH 8.0 and 200 mM NaCl, to remove the maltose. Protein concentration for the *in vivo* assays was determined by Bradford assay and BSA was used to plot the standard curve.

Relative expression of LeishIF-4E1, LeishIF-4E4, Leish4E-IP1 and Leish4E-IP2 in overexpressing cell lines as compared to expression in wild type

Different cell lines overexpressing LeishIF-4E1, LeishIF-4E4, Leish4E-IP1, Leish4E-IP2 and wild type were harvested at mid-log stage. The cells were washed twice with PBS and finally resuspended in 200 µl of PRS plus. Further 200 µl of sample buffer was added and boiled at 100°C for 5 min. Cell extracts were separated by SDS-PAGE and subjected to western analysis using specific antibodies against LeishIF-4E1, LeishIF-4E4, Leish4E-IP1 and Leish4E-IP2.

Growth analysis

L. amazonensis cells overexpressing SBP-tagged LeishIF4E-1, LeishIF4E-3, LeishIF4E-4, Leish4E-IP1, Leish4E-IP2 and control cell line expressing chloramphenicol acetyltransferase (Zilka

et al., 2001) along with WT were cultured as promastigotes at 25°C in M199 medium containing all supplements. All these cells were subjected for growth curve analysis. Cells were seeded at a concentration of $5X10^5$ cells/ml, and the further counted daily during 5 consecutive days. The curves were obtained from three independent repeats.

Generation of specific antibodies against Leish4E-IP2

His-tagged Leish4E-IP2 cloned into the BamHI and Xho1 sites of the pHisParallel 2 expression vector (8). Expression was induced in BL21 cells by IPTG at 30°C. Cells were solubilized and expressed cells. The bacterial cell pellets were resuspended in denaturation buffer (DB, 50 mM Tris:HCl pH 7.4, 300 mM NaCl, 10mM imidazole containing 6M urea) and sonicated X2 for 1.5 minutes using cycles of 10 seconds with 10 seconds intervals). The disrupted cells were spun down by ultra-centrifugation at 15,000 g at 40 min and the supernatant was further purified over Ni_NTA column at 4°C. The column was washed with the DB containing 6M urea at pH 8, and then with DB at pH 6.3. The protein was finally eluted by addition of DB containing 6M urea at pH 4.5. The eluted fraction was loaded on 12% SDS-PAGE and the induced band (slightly stained) was extracted from the gel and used for immunization of New Zealand rabbits, by Adar Biotech, Israel (a commercial company). Two rabbits were each immunized sub-cutaneously with 250 µg emulsified in Complete Freund's Adjuvant, followed by three additional boosts in Incomplete Freund's Adjuvant, in 3 weeks intervals. Rabbits were bled twice 10-14 days following the final boost. Rabbit sera from different bleeds were tested in western analyses.

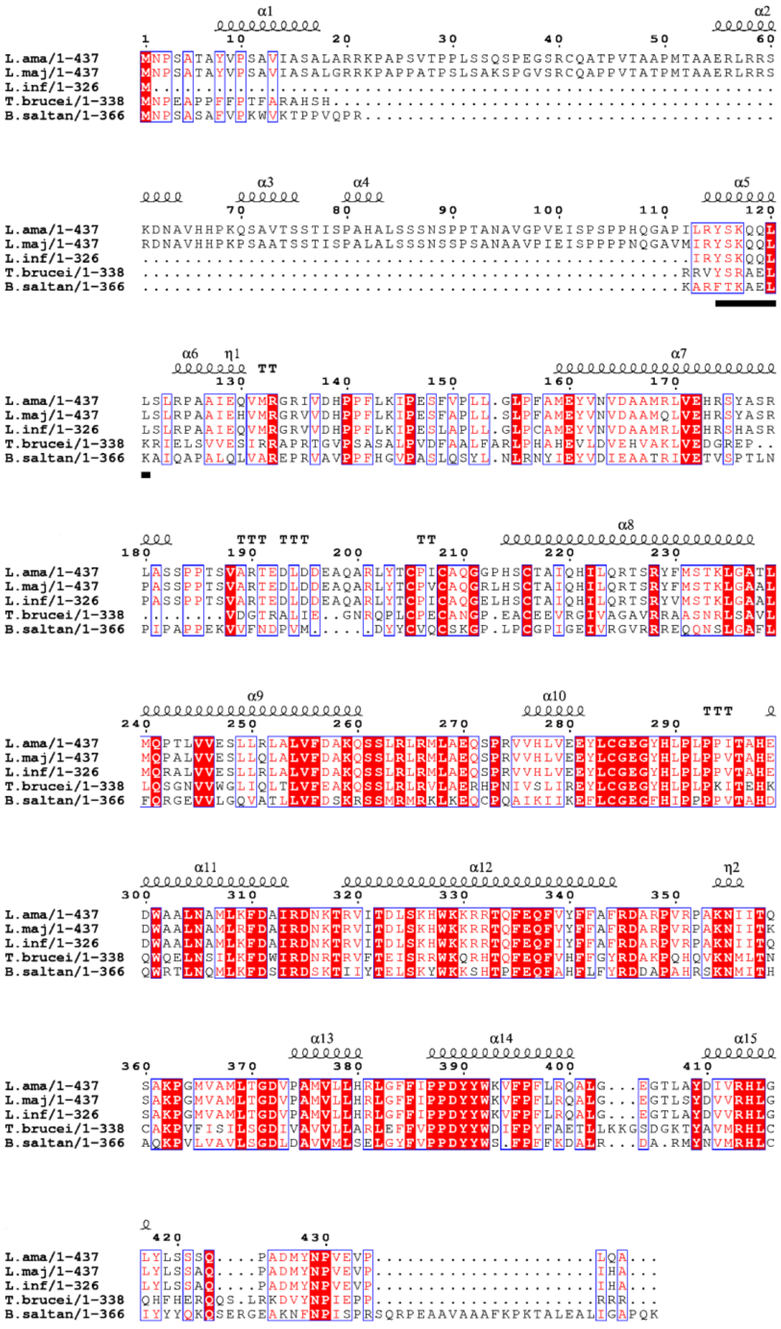

Figure S1. Sequence alignment and secondary structure prediction of Leish4E-IP2 from different *Leishmania* species, from *T. brucei* and *B. saltans.* Sequence alignment was generated using Jalview (2.10.5). The consensus binding motif, Y(X_4)L**Φ**, is underlined. The aligned sequences were derived from *L. amazonensis*, (L. ama, LAMA_000698700); *L. major* (L. maj, LmjF33.0380); *L. infantum*, (L. inf,– LinJ 330010200); *T. brucei* (Tb927.10.11000) and *Bodo saltans* strain *Lake Konstanz* (BSAL_75100)**.** Secondary structure of Leish4E-IP2 was generated using PHYRE[2] V 2. The final alignment with the secondary structure predictions was displayed using the online ESPript 3 tool (http://espript.ibcp.fr/ESPript/ESPript/). White letters over a red background correspond to identical residues while red letters over a white background show conservation. The predicted secondary structures for the *L. amazonensis* Leish4E-IP2 (L. ama, A43530 Scaffold2245 nucleotide positions 442-1755, from http://bioinfo08.ibi.unicamp.br/leishmania/) are indicated as **α**: alpha helices, η: 3_{10}-helix, b: beta-strands, TT: strict b-turns.

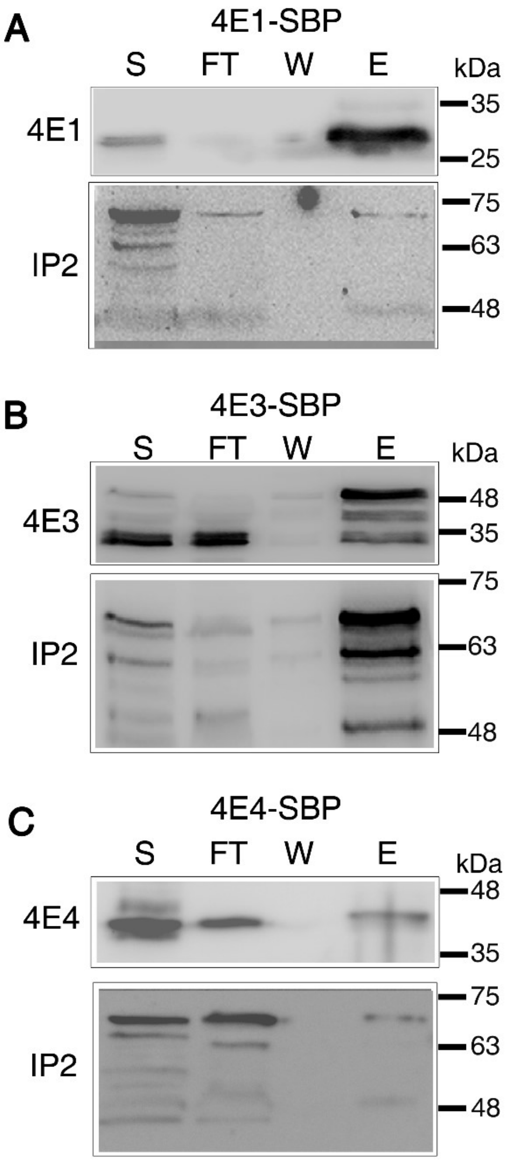

Figure S2. Co-purification of Leish4E-IP2 with LeishIF4E-1, LeishIF4E-3 and LeishIF4E-4 over streptavidin-Sepharose beads. Pull-down experiments were performed with extracts from *L. amazonensis* promastigotes expressing LeishIF4E-1, LeishIF4E-3 and LeishIF4E-4 tagged at their C-termini with SBP. Aliquots from the soluble extract (S, 5%), flow-through (FT, 5%), final wash (W, 50%) and the eluted fraction (E, 50%) were separated by 10% SDS–PAGE gel. Western Blots were performed using specific antibodies against LeishIF4E-1 (4E1), LeishIF4E-4 (4E4), LeishIF4E-3 (4E3) and Leish4E-IP2 (IP2).

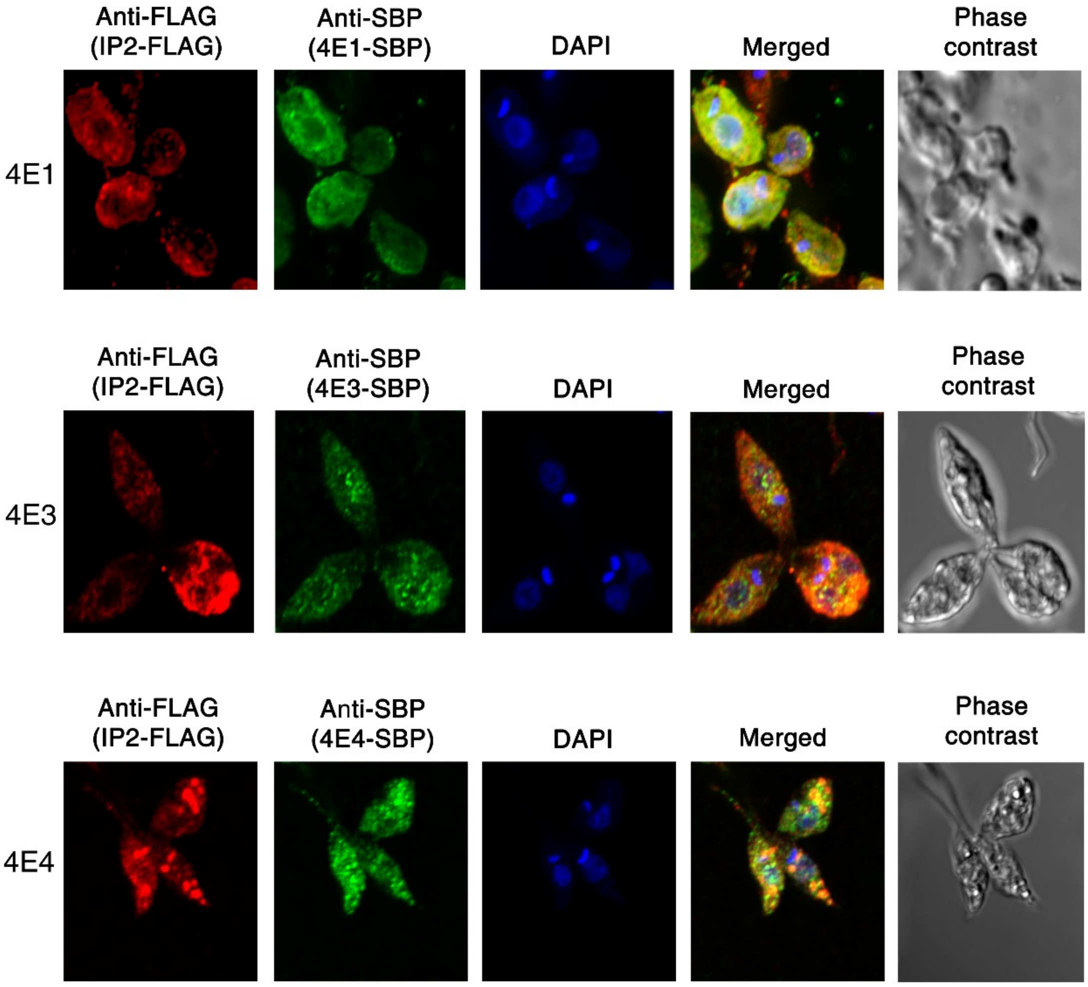

Double cell line	Number of cells analyzed	% Co-localization
IP2-4E1	283	62.0 ± 18.68
IP2-4E3	256	29.3 ± 15.19
IP2-4E4	275	23.0 ± 9.13

Figure S3. Leish4E-IP2 costains with Leish4Es *in situ*. Upper panel: *L. amazonensis* cells expressing FLAG-tagged Leish4E-IP2 and individual SBP-tagged LeishIF4Es (LeishIF4E-1, -3 and -4), were immune-stained with respective primary antibodies (rabbit anti-FLAG and mouse anti-SBP). Secondary antibodies were labeled with a fluorescence tag [Alexa Fluor 488 (green) for the anti-mouse antibodies and DyLight 550 (red) for the anti-rabbit antibodies]. Images were captured in a confocal microscope. **Lower panel:** The Costes-based method for percentage calculation and standard deviation (SD) of co-localization is given (9). Co-localization was calculated for more than 250 cells for each double transgenic cell line.

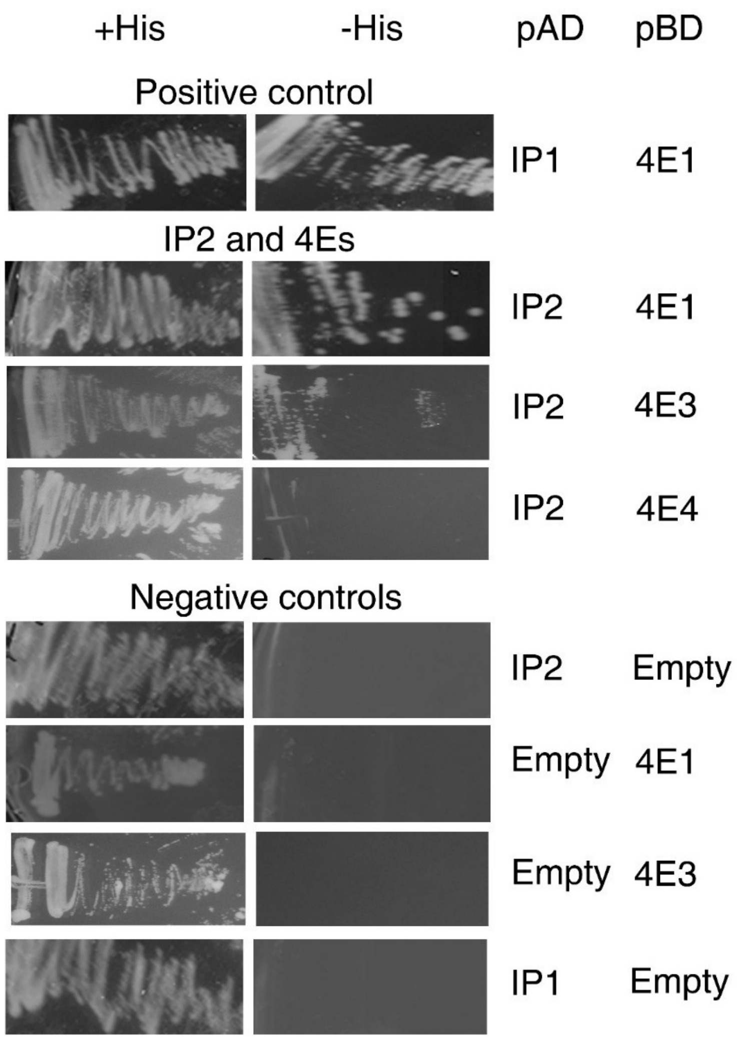

Figure S4. Leish4E-IP2 interacts with LeishIF4E-1 and LeishIF4E-3 in a yeast two hybrid assay. YRG-2 yeast cells were co-transfected with pAD expressing Leish4E-IP2 and pBD plasmids expressing and LeishIF4Es -1, -3 and -4. The interaction between Leish4E-IP1 and LeishIF4E-1 was used as a positive control and empty pAD or pBD plasmids were used as negative controls. Cell growth was examined under restrictive (-His) and permissive (+His) conditions.

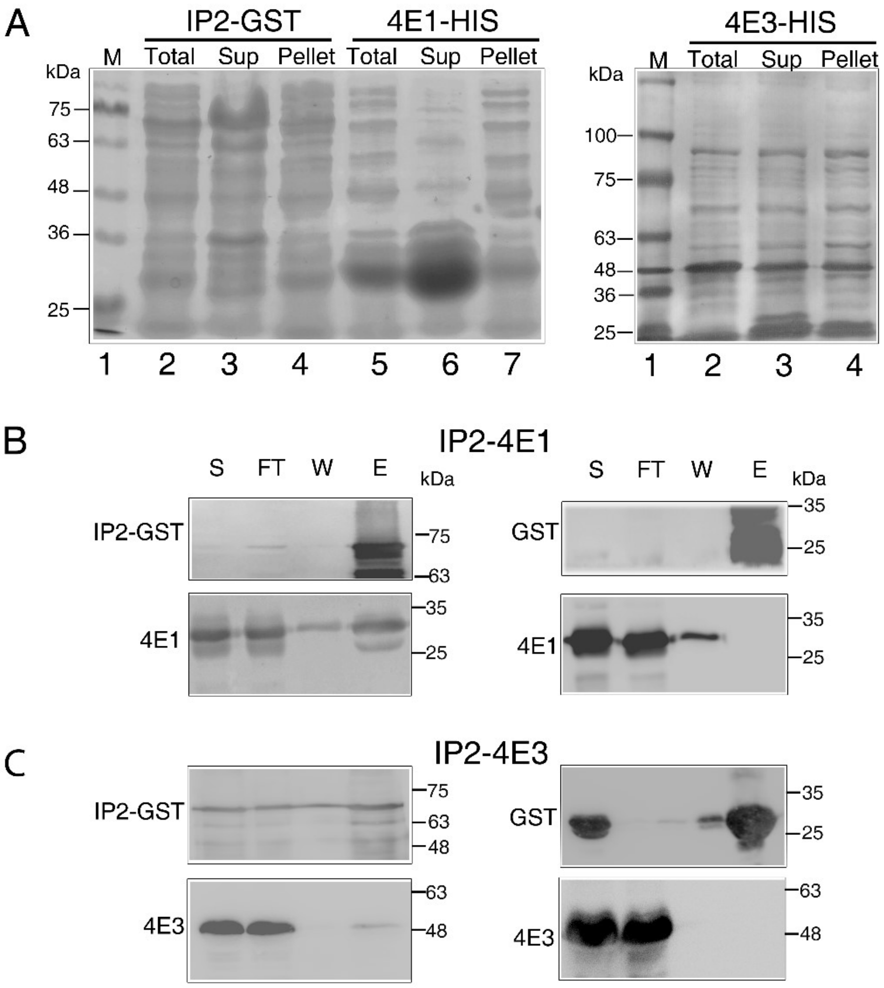

Figure S5. Recombinant Leish4E-IP2-GST interacts directly with recombinant LeishIF4E-1 and LeishIF4E-3. (A) Full-length GST tagged Leish4E-IP2 (left panel, lanes 2-4), and the His-tagged LeishIF-4E1 (left panel, lanes 5-7) and LeishIF-4E3 (right panel lanes 2-4) were expressed in BL-21 cells. Aliquots from the total extracts (Total) the fractionated supernatant (Sup) and the pellet (Pellet) were resolved over SDS-PAGE and further stained with Coomassie blue R-250. **(B-C)** Leish4E-IP2 interacts with LeishIF4E-1 (B) and LeishIF4E-3 (C) *in vitro*. The bacterial lysates (from 200 ml cultures) expressing Leish4E-IP2-GST and GST alone (as a negative control) were bound to Glutathione-Agarose beads for 2 h at 4 °C. The beads were washed and further incubated with 1 ml of bacterial lysate derived from 200 ml bacterial cell cultures expressing His-tagged LeishIF4E-1 or LeishIF4E-3 for 2 h at 4 °C. The beads were washed with 1 ml of cold lysis buffer five times, after which the bound proteins were eluted by boiling the beads with 100 µl SDS sample buffer. Aliquots derived from the supernatant (S, 10%), the flow through (FT, 10%), the wash (W, 25%) and the eluted fractions (E, 25%) were separated over the SDS-PAGE, and further subjected to Western Blot analysis. We used antibodies against LeishIF4E-1 (marked as 4E1, shown in panel B), antibodies against the His-tag (marked as 4E3, shown in panel C) and antibodies against the GST tag (marked as IP2-GST and GST).

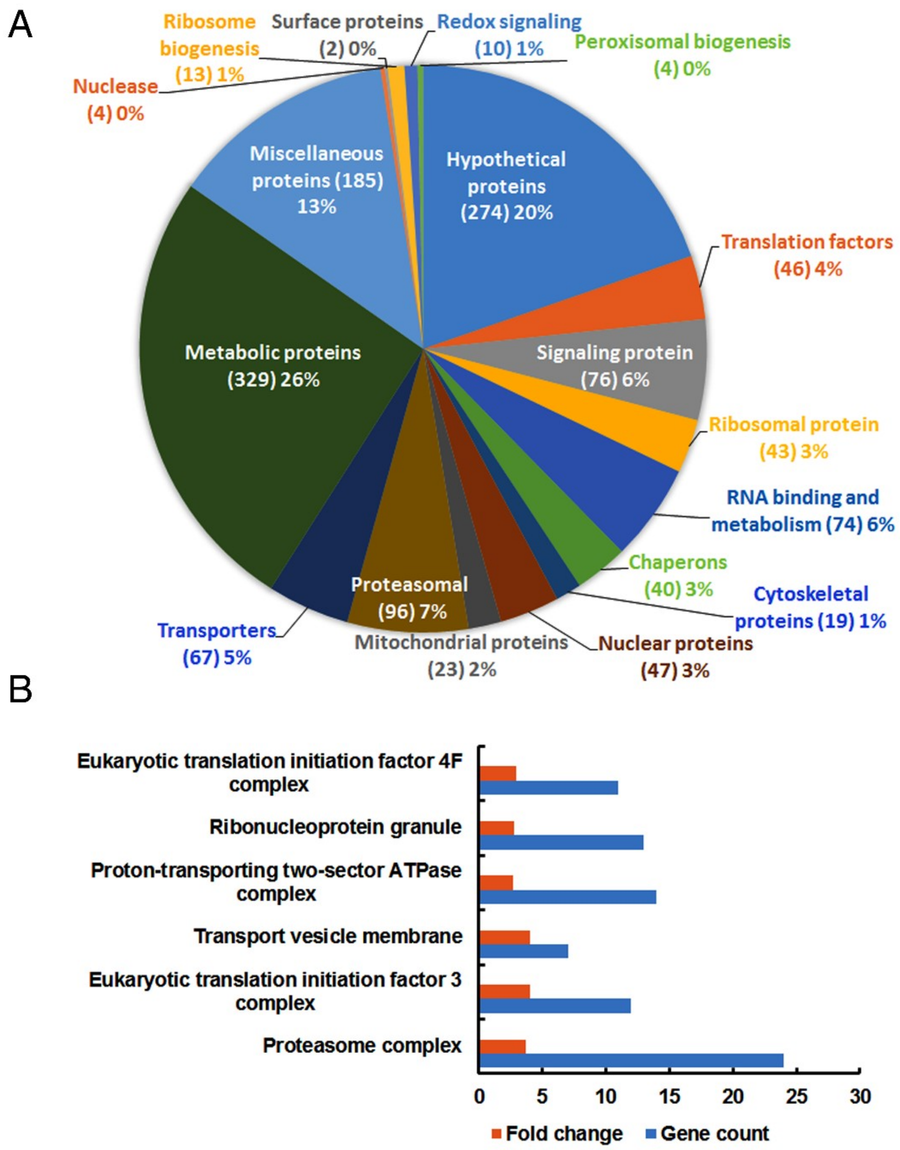

Figure S6. The categorized proteome enriched in Leish4E-IP2-SBP lysates purified over streptavidin-Sepharose, as compared to Luciferase-SBP. The proteome associated with Leish4E-IP2-SBP and Luciferase-SBP was determined by LC-MS/MS analysis, in triplicates. Raw mass spectrometric data were analyzed and quantified using the MaxQuant software and the peptide data were searched against the annotated *L. amazonensis* proteins listed in TriTrypDB. The summed intensities of the peptides that served to identify the individual proteins were used to quantify changes in the proteomic content of specific proteins. Statistical analysis was done using the Perseus software. Proteins that were enriched in the Leish4E-IP2-SBP pull-down by 3 fold ($\log_2$ 1.6) as compared to Luciferase-SBP pull-down, with $p<0.05$ are shown. **(A**) Proteins that were enriched in the Leish4E-IP2-SBP pull-down by 3 fold as compared to Luciferase-SBP pull-down with $p<0.05$, were clustered manually into functional categories. The pie chart represents the summed intensities of enriched protein categories in Leish4E-IP2-SBP pull-down. **(B)** Enriched proteins were classified by the GO enrichment tool in TriTrypDB, based on Cellular components. The threshold for the calculated enrichment of proteins based on their GO terms was set for 2.5 fold, with a $p<0.05$. This threshold eliminated most of the general groups that represented parental GO terms. GO terms for which only a single protein was annotated were filtered out as well. In some cases, GO terms that were included in other functional terms are not shown, leaving only the representative GO term.

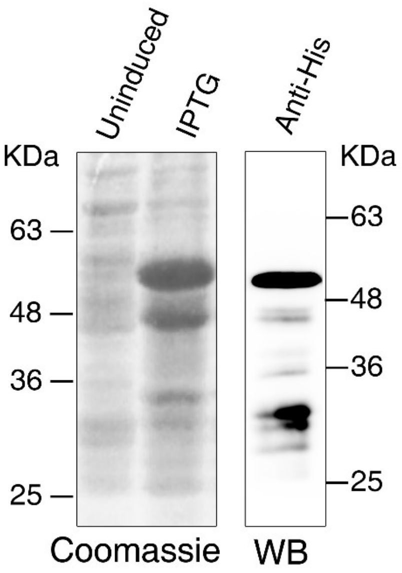

Figure S7. Generation of antibodies against Leish4E-IP2. The open reading frame **(**1314 bp) of LeishIF-4E2 was cloned into the pHIS-parallel vector, fused with a HIS tag, and expressed in BL-21 cells. Left panel: represents the induction of expressed Leish4E-IP2 in BL-21 using 1mM IPTG. Right panel: Expression of LeishIF-4E2 was further confirmed by the specific antibodies against the His-tag.

A

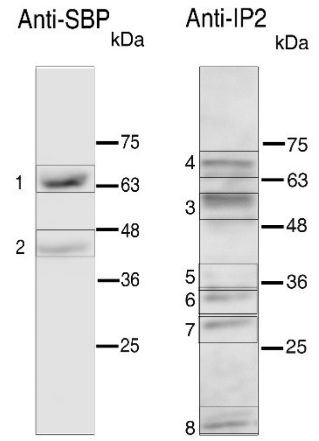

B

Band number	Peptide signature
1	TEDLDDEAQAR, SYASRLASSPPTSVAR
2	TEDLDDEAQAR, VITDLSK, VFPFLR, SAVIASALAR, LASSPPTSVAR, LALVFDAK, DARPVRPAK, QALGEGTLAYDIVR
3	TEDLDDEAQAR, VITDLSK, DARPVRPAK
4	TEDLDDEAQAR, MLAEQSPR
5	TEDLDDEAQAR, MLAEQSPR, SAVIASALAR, LASSPPTSVAR, LGATLMQPTLVVESLLR, VITDLSK, FDAIRDNK
6	TEDLDDEAQAR, VITDLSK, MLAEQSPR, LGATLMQPTLVVESLLR, LASSPPTSVAR,
7	TEDLDDEAQAR, VFPFLR, MLAEQSPR, LGATLMQPTLVVESLLR, LASSPPTSVAR,
8	TEDLDDEAQAR, VITDLSK, VFPFLR, MLAEQSPR, LGATLMQPTLVVESLLR, IVDHPPFLK, LASSPPTSVAR, LALVFDAK, YFMSTK, DARPVRPAK

C

MNPSATAYVPSAVIASALARRKPAPSVTPPLSSQSPEGSRCQATPVTAA
PMTAAERLRRSKDNAVHHPKQSAVTSSTISPAHALSSSNSPPTANAVG
PVEISPSPPHQGAPILRYSKQQLLSLRPAAIEQVMRGRIVDHPPFLKIPE
SFVPLLGLPFAMEYVNVDAAMRLVEHRSYASRLASSPPTSVARTEDLD
DEAQARLYTCPICAQGGPHSCTAIQHILQRTSRYFMSTKLGATLMQPTL
VVESLLRLALVFDAKQSSLRLRMLAEQSPRVVHLVEEYLCGEGYHLPL
PPITAHEDWAALNAMLKFDAIRDNKTRVITDLSKHWKKRRTQFEQFVY
FFAFRDARPVRPAKNIITQSAKPGMVAMLTGDVPAMVLLHRLGFFIPPD
YYWKVFPFLRQALGEGTLAYDIVRHLGLYLSSSQPADMYNPVEVPLQA

Figure S8. **Leish4E-IP2 is susceptible to cleavage**. **A.** *L. amazonensis* lysates were separated over the 10% SDS-PAGE gel and subjected to western analysis using Leish4E-IP2 specific antibodies. Corresponding bands obtained in western analysis were cut from the parallel gel and subjected mass spectrometry analysis. **B.** Represents the peptide identified to corresponding bands. **C** Different peptides identified in Mass spectrometry analysis were highlighted in Leish4E-IP2 protein sequence. The data represent results obtained from three independent experiments. The original Mass spectrometry data are given in Table S5.

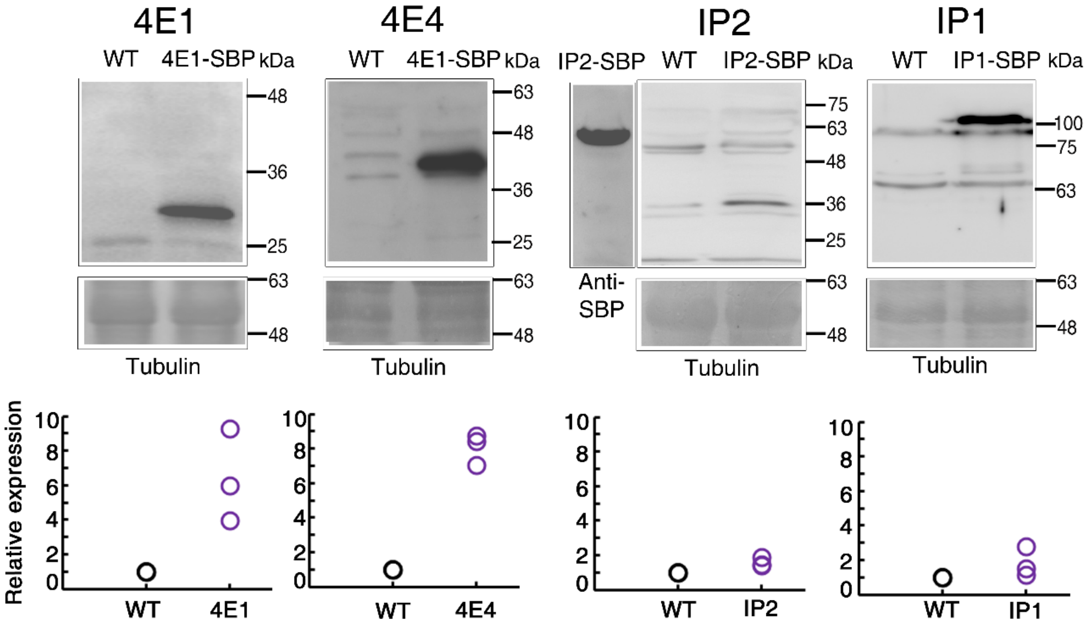

Figure S9. Relative expression of LeishIF-4E1, LeishIF-4E4, Leish4E-IP1 and Leish4E-IP2 in overexpressing cell lines as compared to expression in wild type cells. Upper panel: Different transgenic and wild type cell lysates were resolved over the SDS-PAGE gel, and further subjected to western blot analysis using specific antibodies against LeishIF-4E1, LeishIF-4E4, Leish4E-IP1 and Leish4E-IP2 **Lower panel:** Densitometry analysis of the blot shown in the upper panel.

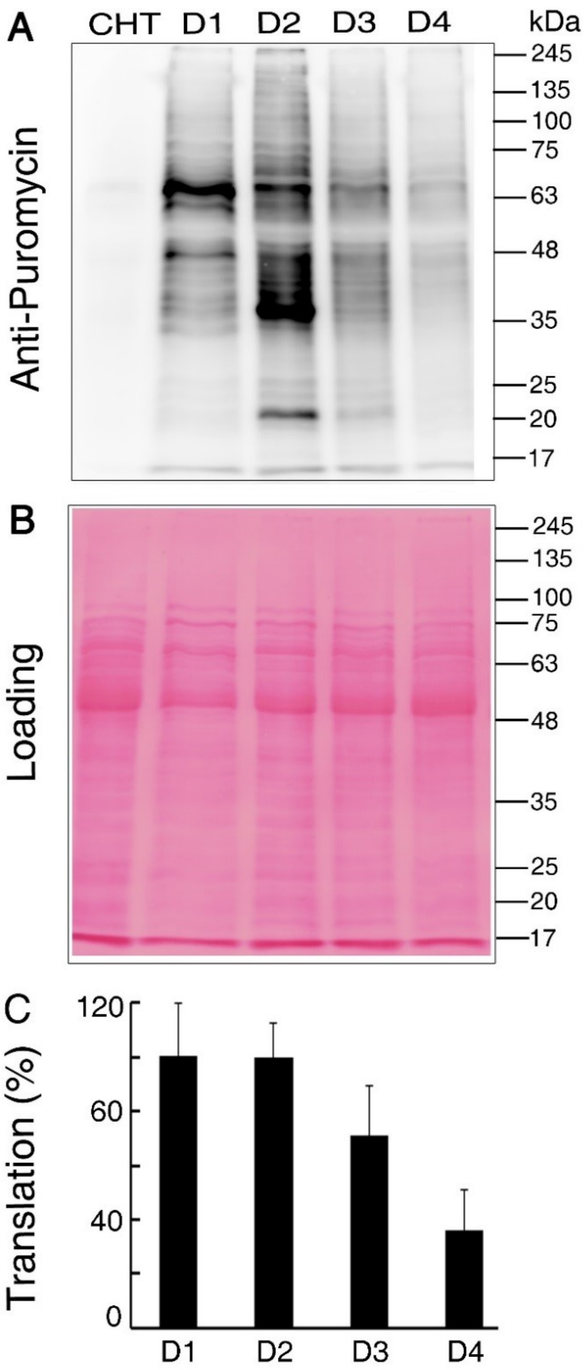

Figure S10. Global translation of wild type promastigotes peaks in early and mid-log cells. (A) Samples from wild type *L. amazonensis* cells taken from different time points along the growth curve were incubated with Puromycin for 30 mins. Whole cell extracts were separated by SDS-PAGE and subjected to western analysis using specific antibodies against puromycin. Cycloheximide (CHX) treatment is shown as control for complete inhibition of translation. **(B)** Shows the Ponceau stain of the blot, to verify equal gel loads. **(C)** Densitometry analysis showing the relative efficiency of translation, shown as precentage of Day 1.

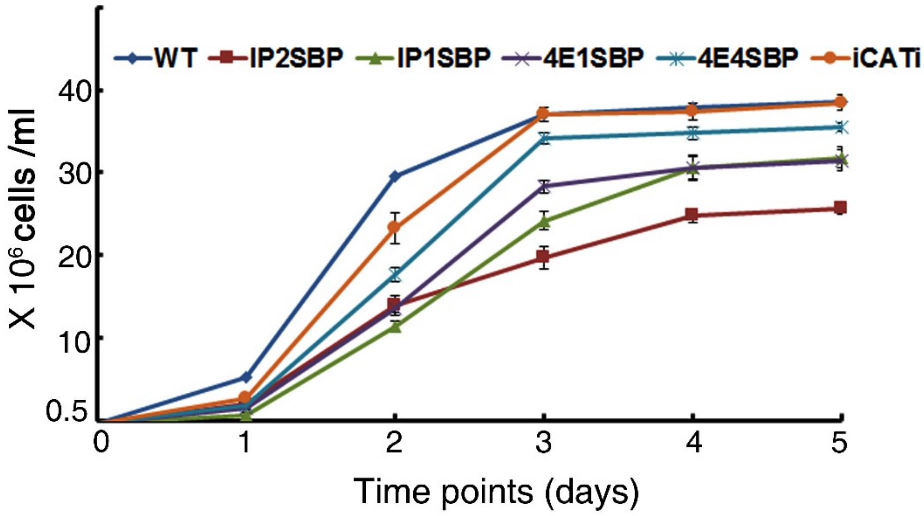

Figure S11. Growth curves of *L. amazonensis* wild type cells, and different transgenic cell lines expressing IP2-SBP, IP1-SBP,4E1-SBP, 4E4-SBP and iCATi (i represents the intergenic region of HSP83 that flanked the CAT reporter gene). Promastigotes were cultured at 25ºC in M199 containing essential supplements**.** Cell counts were monitored daily during 5 consecutive days. The wild type cells are shown in navy blue, cells expressing iCATi are shown in orange, 4E4-SBP in blue, 4E1-SBP in purple, IP1-SBP in green and IP2-SBP in brown.The curves were obtained from three independent assays, error bars are also marked**.**

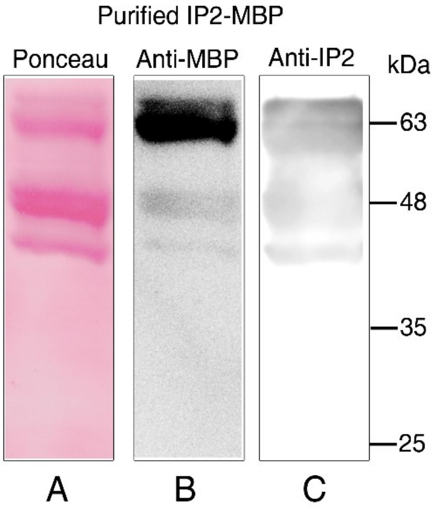

Figure S12. Purification of MBP tagged Leish4E-IP2. The N-terminal fragment of Leish4E-IP2 (1-134) was cloned into the pMBP parallel plasmid (Sheffield et al., 1999). Expression of Leish4E-IP2 (1-134) was induced and the soluble fraction was purified using amylose resin. **A**. Ponceau stain showing the purified Leish4E-IP2 (1-134) N-terminal fragment. **B**. Purified Leish4E-IP2 (1-134) was resolved over the SDS-PAGE gel, and further subjected to western blot analysis, using monoclonal antibodies against MBP. **C**. Purified Leish4E-IP2 (1-134) was resolved over the SDS-PAGE gel and was also subjected to western analysis using antibodies against Leish4E-IP2.

Table S1. List of primers

Name	Sequence	Purpose
IP2FwBamHI	CGCGGATCCATGAATCCGAGCGCCACTG	Clone IP2 in pX-H-target ORF-H-SBP
IP2ReXbaI	GCTCTAGACGCTTGTAAAGGTACCTCAACC	Clone IP2 in pX-H-target ORF-H-SBP
IP2FwBamHI	GGATCCATGAATCCGAGCGCC	Clone IP2 in pX-H-target ORF-H-3X FLAG
IP2Re3XFLAG STOP XbaI	TCTAGATCACTTGTCGTCATCGTCTTTGTAGTCCTTGTCGTCATCGTCTTTGTAGTCCTTGTCGTCATCGTCTTTGTAGTCCGCTTGTAAAGGTAC	Clone IP2 in pX-H-target ORF-H-3X FLAG
IP2FwBamHI	CGCGGATCATGAATCCGAGCGCCACTG	Clone IP2 in pGST
IP2RevXbaI stop	GCTCTAGAGCTACGCTTGTAAAGGTACCTCAACC	Clone IP2 in pGST
Leish4E1FwBamH1	CGCGGATCCATGTCAGCCCCGTCTTCAGTT	Clone 4E1 in pX-H-target ORF-H-SBP
Leish4E1ReXbaI	GCATCTAGATTAGACTAAGACGCCTCGCCGTGC	Clone 4E1 in pX-H-target ORF-H-SBP
Leish4E4FwBamH1	CGCGGATCCATGAACCCCAACGCCACGGA	Clone 4E4 in pX-H-target ORF-H-SBP
Leish4E4ReXbaI	GCATCTAGAGTAGCGCCGACGGTTCTT	Clone 4E4 in pX-H-target ORF-H-SBP
Leish4E3FwBamH1	GCGTTAACTCTAGAACAGAACGTGTGATCG	Clone 4E3 in pX-H-target ORF-H-SBP
Leish4E3ReXbaI	CGAAGCTTGGATCCATGAACCCGTCTGC	Clone 4E3 in pX-H-target ORF-H-SBP
Leish4E1FwEcoRI	CCGGAATTCATGTCAGCCCCGTCTTCAGTT	pBD 4E1
Leish4E1RvSalI	ACGCGTCGACCTAAGACGCCTCGCCGTGCT	pBD 4E1
Leish4E3FwEcoRI	CCGGAATTCATGAACCCGTCTGCCGCTGCA	pBD 4E3
Leish4E3RevEcoRI	CCGGAATTCCTAGAACGTGTGACTGGGCG	pBD 4E3
Leish4E4FwEcoRI	CCGGAATTCATGAACCCCAACGCCACGG	pBD 4E4
Leish4E4RvSalI	ACGCGTCGACTTAGTAGCGCCGACGGTTCTT	pBD 4E4
Leish4E-IP1 FwBamH1	ACTGGATCCATGCCTTCTGTGCGCACC	pAD 4E-IP1
Leish4E-IP1Rev XbaI	ATATCTAGACTAGCGGCGCTGCTGC	pAD 4E-IP1
Leish4E-IP2FwEcoRI	CCGGAATTCATGAATCCGAGCGCCA	pAD 4E-IP2
Leish4E-IP2FwEcoRI	CCGGAATTCCTACGCTTGTAAAGGT	pAD 4E-IP1
5'gRNA IP2	gaaattaatacgactcactataggCGCGTCACCGTGGAAAGCGTgttttagagctagaaatagc	CRISPR-CAS9 KNOCK OUT OF IP2
3'gRNA IP2	gaaattaatacgactcactataggTGGATACCCAGCCTCCAGCTgttttagagctagaaatagc	CRISPR-CAS9 KNOCK OUT OF IP2
Upstream forward	GTCCTCCCGCACGCTGAGCAGCGAAATCCAgtataatgcagacctgctgc	Amplify G418 replacement cassette
Downstream reverse	GCTGGGCGCCGAAGCGCACTACGCCCAACAccaatttgagagacctgtgc	Amplify G418 replacement cassette

Table S2. List of antibodies used for western analysis

Specificity	Organism	Source	Manufacturer	Titer
anti-IP2	*L. major*	rabbit	Adar Biotech	1:2000
anti-IP1	*L. major*	rabbit	Adar Biotech	1:1000
anti-4E1	*L. major*	rabbit	BGU	1:2000
anti-4E4	*L. major*	rabbit	BGU	1:2000
anti-4E3	*L. major*	rabbit	BGU	1:5,000
anti-SBP	Universal	mouse	Millipore	1:10,000
anti-4A1	*L. major*	rabbit	BGU	1:5,000
anti-FLAG	Universal	mouse	Millipore	1:5000
anti-puromycin		mouse	Developmental Studies Hybridoma Bank, University of Iowa	1:1,000
Secondary	Universal	mouse	KPL	1:10000
Secondary	Universal	rabbit	KPL	1:10000

REFERENCES

1. Robert, X. and Gouet, P. (2014) Deciphering key features in protein structures with the new ENDscript server. *Nucleic Acids Res*, **42**, W320-W324.
2. Sheffield, P., Garrard, S. and Deresenda, Z. (1999) Overcoming expression and purification problems of RhoGDI using a family of ™Parallel° expression vectors. *Protein Expr Purif*, **15**, 34-39.
3. Zinoviev, A., Leger, M., Wagner, G. and Shapira, M. (2011) A novel 4E-interacting protein in *Leishmania* is involved in stage-specific translation pathways. *Nucleic Acids Res*, **39**, 8404-8415.
4. Cox, J. and Mann, M. (2008) MaxQuant enables high peptide identification rates, individualized ppb-range mass accuracies and proteome-wide protein quantification. *Nature Biotechnol*, **26**, 1367.
5. Aslett, M., Aurrecoechea, C., Berriman, M., Brestelli, J., Brunk, B.P., Carrington, M., Depledge, D.P., Fischer, S., Gajria, B. and Gao, X. (2009) TriTrypDB: a functional genomic resource for the *Trypanosomatidae*. *Nucleic Acids Res* **38**, D457-D462.
6. Krey, J.F., Wilmarth, P.A., Shin, J.-B., Klimek, J., Sherman, N.E., Jeffery, E.D., Choi, D., David, L.L. and Barr-Gillespie, P.G. (2013) Accurate label-free protein quantitation with high-and low-resolution mass spectrometers. *J Proteome Res*, **13**, 1034-1044.
7. Tyanova, S., Temu, T., Sinitcyn, P., Carlson, A., Hein, M.Y., Geiger, T., Mann, M. and Cox, J. (2016) The Perseus computational platform for comprehensive analysis of (prote) omics data. *Nat Methods*, **13**, 731.
8. Sheffield, P., Garrard, S. and Derewenda, Z. (1999) Overcoming expression and purification problems of RhoGDI using a family of “parallel” expression vectors. *Protein expression and purification*, **15**, 34-39.
9. Costes, S.V., Daelemans, D., Cho, E.H., Dobbin, Z., Pavlakis, G. and Lockett, S. (2004) Automatic and quantitative measurement of protein-protein colocalization in live cells. *Biophys J*, **86**, 3993-4003.
